# Psychosexual Functioning of Cognitively-able Adolescents with Autism Spectrum Disorder Compared to Typically Developing Peers: The Development and Testing of the Teen Transition Inventory- a Self- and Parent Report Questionnaire on Psychosexual Functioning

**DOI:** 10.1007/s10803-017-3071-y

**Published:** 2017-03-16

**Authors:** Linda P. Dekker, Esther J. M. van der Vegt, Jan van der Ende, Nouchka Tick, Anneke Louwerse, Athanasios Maras, Frank C. Verhulst, Kirstin Greaves-Lord

**Affiliations:** 1 Department of Child and Adolescent Psychiatry/psychology, Erasmus MC-Sophia, Wytemaweg 8, Room KP 2881, 3015 CN Rotterdam, The Netherlands; 2 Yulius Academy & Yulius Autism, Yulius, Mental Health Organisation, Dennenhout 1, 2994 GC Barendrecht, The Netherlands

**Keywords:** Psychosexual, Autism spectrum disorder, Adolescence, Comprehensive measure, Typically developing

## Abstract

To gain further insight into psychosexual functioning, including behaviors, intrapersonal and interpersonal aspects, in adolescents with Autism Spectrum Disorder (ASD), comprehensive, multi-informant measures are needed. This study describes (1) the development of a new measure of psychosexual functioning in both parent- and self-reports (Teen Transition Inventory; TTI) covering all three domains of psychosexual functioning (i.e. psychosexual socialization, psychosexual selfhood, and sexual/intimate behavior). And (2) the initial testing of this instrument, comparing adolescents with ASD (*n* = 79 parent-report; *n* = 58 self-report) to Typically Developing (TD) adolescents (*n* = 131 parent-report; *n* = 91 self-report) while taking into account gender as a covariate. Results from both informants indicate more difficulties regarding psychosexual socialization and psychosexual selfhood in the ASD group. With regard to sexual/intimate behavior, only parents reported significantly more problems in adolescents with ASD.

**Electronic supplementary material:**

The online version of this article (doi:10.1007/s10803-017-3071-y) contains supplementary material, which is available to authorized users.

In the past few years, psychosexual functioning in adolescents with ASD has become an increasingly studied topic of research (e.g. Dewinter et al. 2014, 2016; Ginevra et al. 2015; Gougeon 2010; Kellaher 2015; Mehzabin and Stokes 2011). Psychosexual functioning covers not only sexual behaviors, but also interpersonal (i.e. psychosexual socialization; for example relationships) and intrapersonal (i.e. psychosexual selfhood) dimensions (Dewinter et al. 2013). Although not necessary, the interpersonal and intrapersonal elements may be the basis for a healthy overall psychosexual functioning (O’Sullivan et al. 2007). For example, having a crush, or developing a relationship with someone and having sexual desires, may be the foundation for the development of partnered sexual behavior (Hearn et al. 2003), although this has not be studied in individuals with ASD. Much research into psychosexual functioning in individuals with ASD has focused on problematic aspects, using primarily parent or clinician report. For example; autistic traits have been related to excessively thinking about sex, public masturbation, stalking and sexual offenses (i.e. both as victim and as perpetrator) (e.g. Dekker et al. 2014; Dewinter et al. 2013; Hellemans et al. 2007; Sevlever et al. 2013; Stokes and Kaur 2005; t Hart-Kerkhoffs et al. 2009). However, there is also research into psychosexual functioning in individuals with ASD which has focused on the typical aspects, using mostly self-report. This research has shown that individuals with ASD have sexual needs and desires, are interested in romantic relationships and have similar socially accepted experiences and behaviors compared to other groups (Dewinter et al. 2014; Gilmour et al. 2012; Hénault 2006; Kellaher 2015; Stokes et al. 2007). It is valuable to expand the acquired knowledge on psychosexual functioning in adolescents with ASD and obtain information from multiple informants in all domains of psychosexual functioning. This can provide a more well-rounded and multi-dimensional perspective on psychosexual functioning in individuals with ASD.

Psychosexual functioning can be divided into three domains: psychosexual socialization (interpersonal), psychosexual selfhood (intrapersonal), and sexual/intimate behavior (Dewinter et al. 2013; Tolman and McClelland 2011). Psychosexual socialization includes the interaction with social contexts (e.g. peers, parents, siblings, and media) in which individuals learn about and experience relationships and sexuality. Psychosexual selfhood relates to the intrapersonal functioning including self-esteem, self-perceived competence, and knowledge. Sexual/intimate behavior includes a continuum of sexualized behaviors and experiences, ranging from typical, age-appropriate behaviors to atypical, inappropriate or even illegitimate behaviors. Unfortunately, in the current literature on individuals with ASD, most of the studies on (psycho)sexual socialization have primarily used parent or caregiver report, psychosexual selfhood is generally understudied, and when self-report was used the focus was solely on sexual behavior (Dewinter et al. 2013; Fenton et al. 2001).

The usage of either self-report or other-report may be related to issues of assumed insight and knowledge regarding the topic of research and/or the capacity to report reliably on the topic. At least in Typically Developing (TD) individuals (Fenton et al. 2001), typical and socially accepted aspects of psychosexual functioning are often considered fairly private. Therefore, others are thought to not be optimal informants about these topics, which may have led to primarily using self-report. The limited use of self-report in the other domains in the current literature on psychosexual functioning in ASD (see for reviews Byers et al. 2013; Dewinter et al. 2013; Gougeon 2010) may be because the reliability and validity of self-report in individuals with ASD has been questioned (Cederlund et al. 2010; Urbano et al. 2013). Most likely the use of clinician or parent report stems from the assumption that individuals with ASD have little insight into the problematic aspects. However, even with regard to overt topics, such as behavior, a recent study of Dewinter et al. (2016) showed that parents and adolescents do not report identically, implying that the type of informant may influence the results and thus the conclusions. To conclude, parents or clinicians may be better to report on some issues requiring difficult social insight. However, adding self-report also provides meaningful information, namely the adolescents own perspective on their psychosexual functioning (i.e. psychosexual socialization and psychosexual selfhood) and their private experiences (i.e. sexual/intimate behaviors) (Lerner et al. 2012). When it comes to psychosexual functioning in individuals with ASD, the use of multiple informants seems valuable to get a more complete picture as well as insight into how the informant is experiencing psychosexual functioning.

Besides the limited use of multiple informants, few studies have directly compared the psychosexual functioning of adolescents with ASD to a TD control group. This has limited the ability to compare psychosexual functioning in the two groups and investigate the influence of ASD on psychosexual functioning. A comprehensive measure designed for multiple informants which gives insights into the functioning on all three domains of psychosexual functioning used in both an ASD and TD group, would provide data to compare the two groups, thus giving insight into the potential differences and difficulties related to ASD.

As psychosexual functioning is a complex, multifaceted concept (World Health Organization 2006), this in turn has complicated the development of a questionnaire which covers all domains and is suitable for multiple informants. Many currently existing questionnaires only cover one domain of psychosexual functioning or are not suitable for multiple informants. Therefore, in an attempt to add to the growing body of research on psychosexual functioning in adolescents with ASD, we developed the Teen Transition Inventory (TTI; for a detailed description see Measurements) for the purpose of this study. In the development of this tool, we adopted the broad definition of psychosexual functioning described before, covering the three domains: psychosexual socialization, psychosexual selfhood, and sexual/intimate behavior (Dewinter et al. 2013; Tolman and McClelland 2011). Items regarding the first domain, i.e. psychosexual socialization, inquire for example into the skills related to social and intimate contact, openness regarding intimacy and dealing with boundaries. This domain distinguishes between more basic social skills (e.g. Friendship Skills and Social Acceptance) that form a prerequisite for more complex intimate social skills (e.g. Romantic skills). Existing questionnaires on social qualities of individuals with ASD, e.g. the Social Responsiveness Scale (Constantino and Gruber 2007) focus mainly on known autistic difficulties in social relations. In the TTI the focus is more on typical basic and complex intimate social skills that are the basis for healthy psychosexual functioning. Items regarding the second domain, psychosexual selfhood, seek to get information for example about sexual preference, body image, the level of self-esteem, desires, self-perception, and psychosexual knowledge. Questions in the third domain, sexual/intimate behavior, entail the behavioral repertoire that people have with regard to sexuality both online and offline, covering appropriate and inappropriate sexualized behavior (including the amount and type of behaviors), and the age of onset. Moreover, we developed the TTI in both a self-report and a parent-report version. However, in line with previous research, some items are only posed in the TTI of one informant, as we expected the other informant to not be able to reliably answer some items (for example items regarding appropriately dealing with boundaries was only asked to parents and general self-esteem was only part of the self-report TTI).

In the current paper we describe the development of the TTI. Which was specifically developed for the purpose of this study, to allow us to compare all aspects of psychosexual functioning of cognitively-able adolescents with ASD to TD adolescents. In addition, the initial testing of the TTI is discussed, involving (1) the pilot testing of the internal consistency of the theoretically constructed scales regarding psychosexual functioning, and (2) examining whether the scales and items of the TTI distinguish between cognitively-able adolescents with ASD versus their TD peers. Based on previous literature, we hypothesized that cognitively-able adolescents with ASD compared to their TD peers have less psychosexual socialization (e.g. less social acceptance and less adequately dealing with boundaries), poorer psychosexual selfhood (e.g. poorer body image, less confidence, less perceived social competence, lower self-esteem and less knowledge despite equal social, romantic and sexual desires), and display more inappropriate sexualized behaviors, but have similar experiences with appropriate sexual/intimate behaviors (e.g. Brown-Lavoie et al. 2014; Dewinter et al. 2014; Kasari et al. 2011; Nichols and Blakeley-Smith 2009; Stokes et al. 2007). Given the fact that previous studies only used one informant (i.e. either parent or self-report), we were particularly interested to see whether these hypotheses would be confirmed using both parent- and self-report.

## Method

### Sample & Procedure

#### Between 2011 and 2012, Data for this Study were Collected from an ASD Group and a TD Group

The ASD group (*n* = 79) was selected from a larger clinical sample from the outpatient’s Department of Child and Adolescent Psychiatry/psychology of the Erasmus MC—Sophia in Rotterdam, the Netherlands who were participating in a follow-up study. In the initial study between July 2002 and September 2004, 503 children were referred for psychiatric evaluation. Of the 503 referrals, 234 children were eligible to be included in the follow-up study approximately 7 years later due to their social and/or communication problems during the first study (Louwerse et al. 2015). Of the 234 children, 104 (42.3%) children had a best-estimate ASD diagnosis, which was based on the ADI-R and ADOS (see ASD diagnostic procedure below) and thus received the TTI to participate in this part of the study. 79 (76.0%) parents of the 104 individuals with a best-estimate ASD diagnosis returned the Teen Transition Inventory (TTI) parent-report. The parents who did return the TTI did not significantly differ from the group of parents who did not return the TTI with regard to the adolescent’s age (*t*(102) = 1.50, p = .14), intelligence (*t*(75) = −0.39, p = .70) or gender (χ^2^(1, *n* = 104) = 0.61, p = .44). Of the 79 individuals with parent-report, we received 58 (73.4%) self-report TTI’s. The group without self-report data did not differ significantly from the group with self-report data on age (*t*(77) = 0.68, p = .50), intelligence (*t*(59) = −1.43, p = .16), or gender (χ^2^(1, *n* = 79) = 0.46, p = .50), nor on any of the parent-reported psychosexual scales (for a full description of the scales see measurements: Teen Transition Inventory). The mean age of the adolescents with ASD (*n* = 79) was 16.79 years (range 13–21, *SD* = 2.05) and the majority was male (86%). Please see Table 1 for all descriptive characteristics of the ASD group.

**Table 1:** Characteristics of participants

	ASD				TD				
	N (%^a^)	Mean	SD	Range	N (% ^a^)	Mean	SD	Range	p
Parent-report TTI	79 (100%)				131 (100%)				
Self-report TTI	58 (73.4%)				91 (69.5%)				
Male	68 (86.1%)				60 (45.8%)				< 0.001
Age		16.79	2.05	13–21		16.31	1.59	13–20	0.08
Intelligence		98.56	17.88	54–135		100.00	15.00	64 – 152	0.57
Tanner stage		4.32	0.94	1–5		4.42	0.72	2–5	0.41
ADI-R Diagnostic	78* (99%)	36.80	11.59	4–59					
ADI-R Current	75*^†^ (95%)	24.72	8.10	10–44					
ADOS CSS	72 (91%)	5.88	2.39	1– 10					

*One did not participate with ADI-R interview

* ASD* autism spectrum disorder; *TD* typically developing; *TTI* Teen Transition Inventory; *ADI-R* autism diagnostic interview-revised; *ADOS CSS* autism diagnostic observation schedule calibrated severity score

^†^Two participants were non-verbal, and for one participants only the diagnostic score was available due to a clinical evaluation process at another facility

^a^Percentages are based on sample size of parent-report

The TD sample was drawn from a Dutch general population study (*n* = 1710) (Evans et al. 2012; Louwerse et al. 2013; Tick et al. 2008) from which 326 individuals were eligible to participate as they were between 12 and 21 years old (Evans et al. 2012). Of the 326 adolescent and their parents who were contacted, 153 (47%) returned the parent-report and 113 (35%) the self-report. To ensure the TD group would be without autistic traits, we additionally excluded individuals if they had elevated autistic traits (*n* = 22) as assessed with the Autism Quotient (AQ; Baron-Cohen et al. 2001). The final number of included participants; i.e. those who returned the TTI and without autistic traits on the AQ, was 131 (40% of the originally selected adolescents and 86% of those who returned the TTI). Of the 131 participants with parent-report, 91 (=69.5%) self-report TTIs were returned. Those without self-report data did not differ significantly from the group with self-report data on intelligence (*t*(118) = −0.66, p = .51), or gender (χ^2^(1, *n* = 131) = 1.96, p = .16) and on seven of the nine parent-reported psychosexual scales (see Measurements: Teen Transition Inventory). However the group with self-report data was significantly younger (*M* = 16.12, *SD* = 1.47) than the group without self-report data (*M* = 16.73, *SD* = 1.77; *t*(129) = 2.03, p = .04). In addition, the group with self-report data had higher parent-reported family openness regarding sexuality (*M* = 1.22, *SD* = 0.42) than those without self-report (*M* = 0.98, *SD* = 0.40; *t*(126) = −3.04, p < .01) and less parent-reported sexual experiences (*M* = 0.48, *SD* = 0.37) than those without self-report (*M* = 0.64, *SD* = 0.36; *t*(120) = 2.15, p = .03). The mean age of the TD sample (*n* = 131) was 16.31 years (range 13–20, *SD* = 1.59) and 46% of the sample was male. Table 1 provides descriptive information on the TD group.

To assure we were able to make a viable comparison regarding psychosexual functioning between the ASD and TD group, we ascertained whether the groups differed in characteristics such as age, intelligence, physical development and gender, to potentially control for these variables in the main analyses (for further details, please see section Statistical analyses). The results of these comparisons are displayed in Table 1. The study was approved by the Medical Ethical Review Committee of the Erasmus MC. All parents and adolescents gave informed consent.

### ASD Diagnostic Procedure

To obtain a best-estimate clinical diagnosis of ASD, the Autism Diagnostic Interview Revised (ADI-R; Rutter et al. 2003) was performed with parents, and the Autism Diagnostic Observation Schedule (ADOS; Lord et al. 2000) was administered to the adolescents. The ADI-R and ADOS were administered by examiners who had completed the research-training and had achieved sufficient reliability for administration and coding. Based on age and language capability, module 4 of the ADOS was primarily used, although for 6 participants the ADOS module 3 was used. Both examiners reviewed DSM-IV-TR criteria of ASD (i.e. Pervasive Developmental Disorder) and together obtained a consensus diagnosis (Falkmer et al. 2013). Although 32% of the ASD cases (n = 23) did not meet the diagnostic cut-off of the ADOS, these cases received a best-estimate clinical diagnosis based also on the information obtained during the ADI-R.

### Measures

#### Teen Transition Inventory—Measurement Development

##### General Description of the Teen Transition Inventory

The Teen Transition Inventory (TTI) was primarily developed for the purpose of this study, which was to get a better insight in the psychosexual functioning (i.e. psychosexual socialization, psychosexual selfhood and sexual/intimate behavior) of adolescents with ASD compared to TD adolescents. The TTI is based and expanded upon previous research in the field of psychosexual functioning in individuals with ASD (see below). As psychosexuality may be experienced as a rather delicate topic, and is probably related to more general aspects of social and physical development, it was decided to structure the TTI in such a way (i.e. build in subheadings) that it starts with less intimate questions on more general adolescent transition issues (such as the experience of the transition to secondary school and building new friendships, physical development, and leisure activities), followed by the more personal questions on psychosexual functioning (i.e. the bulk of the questionnaire, such as building romantic relationships). As such, more intimate questions regarding intimate relations build on similar questions regarding friendship relations. In the section ‘Psychosexual functioning in the Teen Transition Inventory’ a more in-depth description of the psychosexual domains of the TTI is provided.

The TTI is aimed at individuals between the ages of 12 and 21 years old. To obtain multiple perspectives, the TTI consists of a parent-report version and a self-report version. The parent-report TTI and self-report TTI have considerable overlap in items and thus scales (see Table 2). However, the parent-report TTI includes additional items and scales on topics that parents were considered to be better able to adequately judge (e.g. their child’s psychosexual knowledge and the extent to which their child deals appropriately with boundaries) and thus reliably report on. The self-report TTI includes additional items and scales compared to the parent-report TTI regarding very private and subjective matters (e.g. age of onset of sexual/intimate behavior and self-perceived social competence). In total, the parent-report TTI consists of 148 items and the self-report TTI of 205 items. All scorings of the items are based on information of the last six months to the current state of the adolescent. Most of the items of the TTI are scored on a 3-point scale of ‘Not at all true’; ‘Somewhat or Sometimes true’ and ‘Definitely or Often true’, with the exception of a minority of items (e.g. age of onset and behaviors that can only either be displayed or not, i.e. yes/no answering format). Both the parent-report and self-report take approximately 1 h to complete. The TTI is available upon request (in Dutch, English, Greek and Spanish).

**Table 2:** Teen Transition Inventory (TTI) domain and topical structure

Psychosexual functioning domains	Topic	Parent-report TTI	Self-report TTI
Psychosexual socialization	Friendship skills	Scale of 5 items, e.g. child is good at making friends	Scale of 5 items, e.g. I am good at making friends
	Social acceptance by peers	Scale of 3 items, e.g. child is part of a group of friends	Scale of 6 items, e.g. I am part of a group of friends
	Perceived romantic competence	Not assessed in parent-report TTI	Scale of 3 items, e.g. When I’m in love with someone, I do not know what to do
	Personal openness about intimacy	Not assessed in parent-report TTI	Scale of 3 items, e.g. I discuss my feelings and/or questions about intimacy/sexuality with my parents
	Family openness about intimacy	Scale of 4 items, e.g. in our family we discuss sexuality	Not assessed in self-report TTI
	Adequately dealing with boundaries	Scale of 8 items, e.g. child is able to recognize other people’s boundaries regarding social relationships in general	Not assessed in self-report TTI
Psychosexual selfhood	Sexual preference	*N* = 4 separate items, e.g. I notice a sexual preference in my child; my child has dated a girl; my child has been in love with a boy. The latter recoded to heterosexual love interest; homosexual love interest; heterosexual dating experience; homosexual dating experience	N = 2 separate items, e.g. I have been in love with a girl; I have dated a boy. Recoded to heterosexual love interest; homosexual love interest; heterosexual dating experience; homosexual dating experience
	Body image	Scale of 3 items, e.g. child is satisfied with his/her body	Scale of 7 items, e.g. I am satisfied with the way I look
	Perceived social competence	*N* = 2 separate items, e.g. my child is uncomfortable when he/she is around a group of teenagers	Scale of 12 items, e.g. Around other people I lose my confidence
	Social desires	*N* = 1 separate items, e.g. My child has a need for a best friend	*N* = 10 separate items, e.g. I think it is important my best friend is funny
	Romantic desires	Not assessed in parent-report TTI	*N* = 9 separate items, e.g. I think it is important a (future) partner is funny
	Amount of sexual desires	Not assessed in parent-report TTI	Scale of 6 items, e.g. I want to French kiss; I want to have intercourse; I fantasize sometimes about being intimate?
	Self-esteem	Not assessed in parent-report TTI	Scale of 12 items, e.g. I am happy with myself as a person
	Psychosexual knowledge	Scale of 9 items, e.g. child knows what precautions to take to avoid pregnancy	Not assessed in self-report TTI
Sexual/intimate behavior	Amount of sexual behavior	Scale of 3 items, e.g. my child has had intercourse	Scale of 5 items, e.g. I have had intercourse
	Types of intimate or sexual behavior and experiences	*N* = 24 separate items, e.g. My child has been in love with a celebrity; When in love my child does not know how to make contact with that person; my child has difficulty being touched by family members or well-known acquaintances	*N* = 26 separate items, e.g.I have been in love with a fictional character; the first time I have contact with the person I am in love with, I tell that person I am in love with him/her; I have had a very unpleasant intimate experience; Do you have a specific physical limitation or any physical problems at the moment which make intimate relations or sexuality more difficult?
	Amount of inappropriate sexualized behavior	Scale of 6 items, e.g. my child touches people in places where the other does not want to be touched; Did your child ever masturbate at inappropriate times, in inappropriate ways or places	Scale of 3 items, e.g. I keep contacting someone, even though that person has indicated he/she does not want any contact with me; I touch people in places where the other does not want to be touched
	Online sexual activity	Scale of 3 items, e.g. my child visits websites that give information about sex	Scale of 7 items, e.g. I visit websites that give information about sex
	Age of onset	Not assessed in parent-report TTI	*N* = 5 separate items, e.g. how old where you the first time you had intercourse

* TTI* Teen Transition Inventory

#### Development of the Teen Transition Inventory

The TTI was developed by a team of researchers and clinicians of Erasmus MC—Sophia and Yulius with input from adolescents with ASD and their parents. The researchers who were involved the development of the TTI, had previous research experience with the assessment of adolescents with ASD and their caregivers. The clinicians involved in the development of the TTI were psychologists, sexologists and psychiatrists, who had specific clinical experience with adolescents with ASD and psychosexual concerns. As a first step, based on the earlier literature on sexuality in individuals with ASD as well as gaps noticed in this research, an initial list of questions, their scoring options, and clustering was constructed by the researchers: i.e. covering the topics of psychosexual socialization, psychosexual selfhood and sexual/intimate behavior (based on Gougeon 2010; Hellemans et al. 2007, 2010; Hénault 2006; Koller 2000; Locke et al. 2010; Nichols and Blakeley-Smith 2009; Realmuto and Ruble 1999; Stokes and Kaur 2005; Stokes et al. 2007; Sullivan and Caterino 2008; t Hart-Kerkhoffs et al. 2009; Tolman and McClelland 2011). In the second step, these items, options and their clustering were reviewed by the clinicians, who suggested some changes in the wording (particularly with regard to taking the wording too literal, which some individuals with ASD do; e.g.Dennis et al. 2001; Martin and McDonald 2004), and also suggested the addition of items; i.e. questions regarding inappropriate love interests (e.g. a teacher, therapist or group-leader). After the suggested changes were made, in a third step this revised version of the TTI was piloted during a pilot study to include the input of 12–18 years old adolescents with ASD and their parents, who also participated in a study on the effects of a psychosexual intervention (Tackling Teenage). Participants (*N* = 30) were asked to fill out the TTI and provide it with their written feedback, including open-ended alternative answering options as well as an open page on which participants could provide their suggestions. Also, oral feedback was welcomed, in case preferred. The use of the TTI in the pilot study was undertaken to specifically evaluate the TTI for use among adolescents with ASD and their parents, to ensure that the questions were relevant, clear and understandable for individuals with ASD and their parents. In addition, the pilot allowed us to examine whether the content, covering rather private topics, was experienced as too personal which could lead to people not filling out questions. The pilot ensured that this was not the case, since all participants returned the TTI fully answered. The feedback uncovered that the content of the TTI was experienced as asking quite personal information, and some found it difficult to think of answers as they never consciously thought about these issues before. Yet no feedback was given that topics should be excluded for the reason of being too personal or that other topics should be included. Remaining feedback varied from suggesting changes in the answering options (i.e. hair growth can never be done, thus the answer option was changed from ‘finished’ to ‘full-grown’) to suggesting shortening of the introductory texts on the topics of the TTI. The feedback of the clients and their parents was used to further improve the TTI. As a final step, the researchers optimized the initial TTI scales based on the state-of-the art literature.

#### Psychosexual Functioning in the Teen Transition Inventory

Psychosexual functioning (i.e. psychosexual socialization, psychosexual selfhood and sexual/intimate behavior) is covered by a total of 81 items in the parent-report TTI and 123 items in the self-report TTI. Table 2 illustrates the scale structure of the TTI divided per domain of psychosexual functioning, and provides examples of items for each subscale (a sample of the TTI is provided in appendix 1a + b). As shown, 48 of the items of the parent-report are clustered into 9 scales, with the remaining 33 items used as ‘stand-alone’ items. Of the self-report 69 of the items are clustered into 12 scales, with the remaining 54 items left as ‘stand-alone’ dichotomous variables. ‘Stand-alone’ items reflect the presence or absence of particular behaviors, experiences or qualities that could not be meaningfully clustered into scales (e.g. My child has been in love with a celebrity; I think it is important that my best friend is funny) and were therefore used dichotomously. Any stand-alone item scored on a 3-point scale, was first dichotomized before further analyses. The categories ‘Somewhat or sometimes true’ and ‘Definitely or often true’ were then collapsed into one category and coded as 1 whereas the category ‘Not at all true’ was coded as 0.

Based on the existing literature and on expert opinion, the scales were formed using items that reflect a particular underlying construct (e.g. Dewinter et al. 2013; Realmuto and Ruble 1999; Tolman and McClelland 2011; Urbano et al. 2013). Scale scores are computed by calculating the mean score of all the items on the scale that were filled out, where a minimum of 60% of the items in the scale have to be filled out. Thus, the scale scores reflect the mean score of the items on the scale, either of the parent or the adolescent. Most scale scores range between 0 and 2 except the scales in the sexual/intimate behavior domain (i.e. ‘Amount of sexual/intimate behavior’; ‘Amount of inappropriate sexualized behavior’ and ‘Online sexual activity’) which range between 0 and 1. As behaviors and experiences are considered to either have or have not occurred, most items on the scale were dichotomous, and thus all items on these scales were used as dichotomous items to compute the mean scale score.

### Putative Covariates

Previous studies have related age, gender, intelligence and physical development (i.e. Tanner stages) to psychosexual functioning and to ASD (e.g. Baron-Cohen and Wheelwright 2003; Beier and Ackerman 2003; Flannery et al. 1993; Halpern et al. 1993; Mandy et al. 2012; Shandra and Chowdhury 2012; Stokes et al. 2007; Vickerstaff et al. 2007; Whitehouse et al. 2011). Therefore, it was important to assess these variables.

As measures for intelligence we used abbreviated versions of the Wechsler intelligence scales. In the ASD sample the Wechsler Abbreviated Scale of Intelligence (WASI; Wechsler 1999) was used. The full 4 score of the WASI (i.e. using all of the 4 subtests) was used as a total IQ score. In the TD sample, two subtests of the Wechsler Intelligence Scale for Children were used, namely vocabulary and block design (WISC; Wechsler 2004). On both subtests a score of 10 reflects average intelligence. A total IQ score was calculated for the TD sample by computing the mean scores, transforming this into z-scores, and then transforming the z-score to a total IQ score with 100 as a mean and 15 as the standard deviation.

The Tanner stages are a staging system which uses schematic drawings of secondary sexual characteristics divided into five standard stages (Marshall and Tanner 1969, 1970). In our study we used the parent-rated Tanner stages. The primary caregiver, in our study mostly the biological mother (86%), indicated on the gender appropriate sketches which stage resembled the physical appearance of her child most. The ratings have shown good reliability and validity and have been used widely (Dorn et al. 1990).

### Statistical Analyses

#### Sample Characteristics

For descriptive purposes, we computed means and standard deviations on age, intelligence, and parent-reported Tanner stages and the frequency of gender for the group with ASD and the TD group. For the ASD group, we also computed descriptive scores on the ADI-R (Rutter et al. 2003) and the severity score on the ADOS (Lord et al. 2000), to provide an indication of the ASD severity in our sample. To investigate which of the putative covariates should be included in the main analyses, we examined whether our groups differed on these variables. For age, IQ and tanner stages this was done by means of independent-samples t tests, gender was compared by means of chi square analyses. In addition, we computed correlations between the potential covariates and all scales of the TTI. An association of the potential covariate with group status (ASD versus TD) as well as an outcome measure resulted in the inclusion of this variable as a covariate in the main analyses (i.e. group comparisons).

The primary aims of the current paper were to describe the development of the TTI and to compare psychosexual functioning of adolescents with ASD to TD adolescents. For this purpose, we firstly examined the internal consistency of the scales and correlations between scales, and secondly explored the differences between adolescents with ASD and TD adolescents with regard to psychosexual functioning using the TTI.

#### Measurement Development: Internal consistency

The internal consistency of the scales of the TTI, which were theoretically constructed based upon the available literature and clinical experience of the team developing the TTI, were checked by means of Cronbach’s alpha. The Cronbach’s alphas were calculated separately for the ASD sample and for the TD sample. Items on the scales were checked to have an item-rest correlation of at least 0.3 in at least one of the samples, which indicates that the item measures the same underlying construct (Field 2013). Items that did not meet this criteria were subsequently either retained or removed from the scale based on their content validity and the effect on the Cronbach’s alpha of the scale. If the deletion of an item let to an improvement of 0.1 or more in the Cronbach’s alpha’s in both samples the item was removed from the scale.

#### Measurement Development: Correlations Between Scales

We also ran correlations between the various scales of both self-report and parent-report. Any perfect or near to perfect correlations (> ± 0.90) were considered for data reduction purposes. If the correlation in combination with content seemed to reflect that the scales measured the same construct, this could indicate a scale could be excluded. In addition, the correlations provide insight in how the different domains of psychosexual functioning interrelate and show the associations between self-report and parent-report .

#### ASD versus TD: Group Comparisons

Group comparisons regarding psychosexual functioning were made using analyses of variance adjusted for any relevant covariate (ANCOVA; i.e. for scale scores) or logistic regression analyses (i.e. for categorical separate item scores). For continuous outcomes, e.g. scores on the scales and age variables, group membership (ASD or TD) was the between-subjects factor. For the categorical outcomes, i.e. the dichotomous separate items, we investigated differences between ASD and TD again including any relevant covariate as predictors. Items which showed a significant difference between the ASD sample and the TD sample were subsequently analyzed for frequencies, to investigate which percentage of the groups had that experience.

Since we ran multiple tests to investigate similarities and differences in psychosexual functioning between the adolescents with ASD versus TD adolescents, we used the Bonferroni correction for multiple testing in the group comparison analyses. In the parent-reported data we ran 42 analyses (9 on the scales and 33 on the dichotomous separate items), therefore the appropriate p-value to control for Type I errors is 0.0012 (is 0.05/42). In the self-reported data we ran 65 tests (12 on the scales and 54 on the dichotomous separate items), therefore the appropriate p-value after the Bonferroni correction is 0.0008 (is 0.05/66).

## Results

### Sample Characteristics

The main characteristics of the ASD and TD samples are shown in Table 1. Only gender significantly differed between the group with ASD and the TD group, in which the ASD sample had significantly more males than the TD sample. Correlations between the scales of the TTI and potential covariates per group showed that some of the potential covariates were significantly related to some of the scales (see Tables 3, 4). However, since gender was the only variable related to both group status and outcome measures, only this variable was included as a covariate in all the main analyses.

**Table 3:** Correlations between covariates and scales in the ASD group

	Self-report												Parent-report								
	Socialization				Selfhood					Behavior			Socialization				Selfhood		Behavior		
	FS	SA	RS	PO	BI	SE	SC	RC	SD	SB	ISB	OS	FS	SA	F.O.	A.B.	BI	PK	SB	ISB	OS
Gender	−0.21	−0.42^†^	−0.06	−0.16	−0.09	−0.37^†^	−0.36^†^	−0.05	−0.23	0.07	−0.00	−0.03	−0.13	−0.24^*^	−0.09	−0.24^*^	−0.08	−0.13	−0.00	0.27^*^	−0.14
Age (parent)	−0.15	−0.11	−0.23	−0.01	0.05	−0.13	−0.36^†^	−0.10	0.07	0.17	−0.01	0.14	0.10	0.03	−0.08	0.22	−0.01	0.17	0.14	−0.20	0.15
Age (self)	−0.16	−0.14	−20	−0.04	0.09	−0.13	−0.35^†^	−0.10	0.06	0.14	−0.01	0.12	0.05	0.12	−0.17	0.04	−0.03	−0.08	0.01	−0.17	0.13
IQ	0.22	0.20	0.20	0.05	0.07	0.17	0.42^†^	0.38	0.40^*^	0.26	0.02	0.08	−0.00	−0.03	0.13	0.32^*^	0.12	0.61^*^	0.23	−0.11	0.11
Tanner (parent)	0.08	−0.05	−0.27	−0.16	0.30^*^	−0.07	−0.13	0.37^*^	0.27	0.17	0.04	0.18	0.27^*^	0.21	0.13	0.26^*^	0.27^*^	0.35^†^	0.32^†^	−0.25^*^	0.17
Tanner (self)	−0.00	0.14	−0.27	−0.03	0.32^*^	−0.04	−0.06	0.12	0.33^*^	0.29^*^	0.05	0.18	0.22	0.29^*^	0.24	0.06	0.40^†^	0.30^*^	0.40^†^	−0.12	0.28^*^

*ASD* autism spectrum disorder; *FS* friendship skills; *SA* social acceptance; *RS* romantic skills; *PO* personal openness; *FO* family openness; *AB* adequate boundaries; *BI* body image; *SE* self-esteem; *SC* perceived social competence; *RC* romantic confidence; *SD* amount of sexual desire; *PK* psychosexual knowledge; *SB* amount of sexual behavior; *ISB* amount inappropriate sexualized behavior; *OS* online sexual activity

* = <0.05

^†^ = <0.01

**Table 4:** Correlations between covariates and scales in the TD group

	Self-report												Parent-report								
	Socialization				Selfhood					Behavior			Socialization				Selfhood		Behavior		
	FS	SA	RS	PO	BI	SE	SC	RC	SD	SB	ISB	OS	FS	SA	FO	AB	BI	PK	SB	ISB	OS
Gender (0 = male)	0.16	−0.03	0.03	0.26^*^	−0.20	−0.26*	−0.06	0.06	−0.25	−0.01	0.05	−0.24^*^	0.20^*^	0.01	0.15	0.01	−0.19^*^	0.01	−0.04	−0.11	−0.34^*^
Age (parent)	−0.03	0.07	0.02	0.24^*^	0.01	0.01	0.08	0.20	0.41^†^	0.46^†^	0.07	0.12	0.03	0.11	0.05	0.24^†^	0.02	0.12	0.50^†^	0.12	0.19^*^
Age (self)	−0.12	0.04	0.00	0.12	0.05	0.01	0.03	0.21	0.41^†^	0.47^†^	0.05	0.06	0.01	0.10	0.03	0.24^*^	−0.10	0.05	0.29^†^	0.04	0.08
IQ	−0.15	−0.05	0.25^*^	−0.26^*^	−0.03	−0.20	−0.17	−0.42^†^	0.05	−0.20	−0.05	0.21	−0.03	0.04	−0.09	−0.05	0.09	−0.06	−0.11	−0.01	0.15
Tanner (parent)	0.03	0.05	0.08	0.05	−0.15	−0.11	0.00	0.12	0.04	0.22^*^	0.11	0.06	0.14	0.11	0.16	0.25^†^	0.04	0.11	0.34^†^	0.04	0.10
Tanner (self)	0.13	−0.05	−0.05	0.02	0.00	0.07	0.11	0.23	0.22	0.22^*^	0.07	0.15	0.16	0.12	0.08	0.23^*^	0.01	0.05	0.32^†^	−0.07	−0.03

*TD* Typically developing. *FS* friendship skills; *SA* social acceptance; *RS* romantic skills; *PO* personal openness; *FO* family openness; *AB* adequate boundaries; *BI* body image; *SE* self-esteem; *SC* perceived social competence; *RC* romantic confidence; *SD* amount of sexual desire; *PK* psychosexual knowledge; *SB* amount of sexual behavior; *ISB* amount inappropriate sexualized behavior; *OS* online sexual activity

* = <0.05

^†^ = <0.01

### Measurement development: Internal consistency

One of the goals of the current study was to pilot test the internal consistency of the TTI scales (see Table 5). For each scale the internal consistency was calculated and the item-rest correlations were checked to see if any item on any scale was below 0.3 in both samples. Based on the number of items in each scale, and the number of participants, five of the nine parent-scales and ten of the twelve adolescent-scales showed good (i.e. >=0.7) internal consistency in at least one of our samples (see Table 5 for the respective Cronbach’s alphas; Field 2013; Kline 1999; Ponterotto and Ruckdeschel 2007). In addition, three of nine parent-scales showed moderate (>0.55) internal consistency (Kline 1999; Ponterotto and Ruckdeschel 2007). Only two scales showed low (i.e. <0.5). internal consistency. One in both parent-report and self-report (i.e. Amount of inappropriate sexualized behavior) and one only in self-report (Personal openness about intimacy).

**Table 5:** Cronbach’s alphas of the psychosexual scales of the TTI

	Parent-report		Self-report	
Name scale	ASD	TD	ASD	TD
Friendship skills	0.79	0.57	0.74	0.52
Social acceptance by peers	0.67	0.74	0.82	0.78
Romantic skills	*n.a*	*n.a*	0.82	0.80
Personal openness about intimacy	*n.a*	*n.a*	0.37	0.51
Family openness about intimacy	0.73	0.75	*n.a*	*n.a*
Adequate boundaries	0.89	0.75	*n.a*	*n.a*
Body image	0.60	0.60	0.62	0.74
Self-esteem	*n.a*	*n.a*	0.70	0.70
Perceived social competence	*n.a*	*n.a*	0.75	0.73
Romantic confidence	*n.a*	*n.a*	0.72	0.63
Amount of sexual desire	*n.a*	*n.a*	0.89	0.86
Psychosexual knowledge	0.93	0.91	*n.a*	*n.a*
Amount of sexual behavior	0.57	0.57	0.81	0.82
Amount of inappropriate sexualized behavior	0.42	0.36	0.26	−0.10
Online sexual activity	0.66	0.58	0.66	0.76

* ASD* autism spectrum disorder; *TD* typically developing; *TTI* Teen Transition Inventory

*n.a*. not applicable, i.e. scale only exists in either the parent-report or the self-report version of the TTI

Based on the analyses to check the Cronbach’s alpha, 15 items (six items on parent-report scales and nine items in the self-report scales) showed an item-rest correlation below 0.3 in both samples. Please see specifications in appendix 2. Although an item-rest correlation below 0.3 may indicate that the item does not belong to the scale, all off the items were retained on their respective scales for two reasons. Firstly, removal of the items resulted in minimal improvements of the Cronbach’s alpha (less than 0.1 in both samples), or even decreased the Cronbach’s alpha. Secondly, as this pilot study is the first using the TTI and the Cronbach’s alpha is dependent on sample size and characteristics, removing the items based on these first results in the current sample seems too premature. Current sample sizes are insufficient to run confirmatory factor analyses, which would have been a better way to study the reliability of the questionnaire. Further research with the TTI is on its way to improve its psychometric underpinnings.

### Measurement Development: Correlations Between Scales

Correlations between the scale-scores of both informants (self-report and parent-report) showed a wide variety of correlational strength (see Table 6). Most of the correlations are non-significant (*n* = 69.3%) and represent negligible (0.00 to ±0.10) or small correlations (0.10 to ±.0.30). Of the correlations that do reach significance (*n* = 117), the majority (*n* = 59 = 50.4%) can be qualified as medium effect (>±0.30) and 24 correlation (20.5%) have large effect (>±0.50) (Field 2013). None of the correlations were perfect or near perfect (>±0.90). Thus, there were no correlations which merited data reduction. The correlations were mostly in the expected directions and between scales that were expected to be closely related (e.g. Social Acceptance and Friendship Skills), both within informant and between informants. Only one scale stood out in this respect, i.e. the Romantic Skills scale, which unexpectedly correlated negatively with the majority of the scales on the TTI (both self-report and parent-report). Correlations between informants showed that, in both the ASD and TD group, on all but the scale of Inappropriate Sexualized Behavior the correlations were significant.

**Table 6:** Correlation matrix between the scales of the TTI

		Self-report												Parent-report								
		Socialization				Selfhood					Behavior			Socialization				Selfhood		Behavior		
		FS	SA	RS	PO	BI	SE	SC	RC	SD	SB	ISB	OS	FS	SA	FO	AB	BI	PK	SB	ISB	OS
Self-report	F.S		0.48^†^	−0.32^†^	0.33^†^	0.17	0.26^*^	0.51^†^	0.39^†^	0.02	0.15	0.11	−0.11	0.39^†^	0.24^*^	0.16	−0.06	−0.02	0.05	0.22^*^	0.15	0.13
	S.A	0.54^†^		−0.34^†^	0.19	0.31^†^	0.43^†^	0.56^†^	0.33^†^	0.13	0.09	−0.06	−0.23	0.39^†^	0.57^†^	0.05	0.30^†^	0.12	−0.02	0.12	0.00	−0.04
	R.S	−0.39^†^	−0.21		−0.29^†^	−0.27^*^	−0.33^†^	-53^†^	−0.57^†^	0.06	−0.29^†^	−0.09	0.04	−0.15	−0.04	−0.25^*^	−0.12	−0.11	0.01	−0.38	−0.03	−0.03
	P.O	0.00	0.08	0.20		0.05	0.10	0.23^*^	0.32^†^	0.20	0.24^*^	0.08	0.09	0.14	0.13	0.11	−0.01	−0.05	0.12	0.24^*^	0.12	0.11
	B.I	0.25	0.26	−0.05	−0.05		0.65^†^	0.48^†^	0.40^†^	0.02	−0.04	−0.11	−0.04	−0.01	0.20	0.05	0.19	0.35^†^	−0.06	−0.06	0.16	0.16
	S.E	0.32^*^	0.58^†^	0.01	−0.00	0.50^†^		0.66^†^	0.54^†^	0.15	0.12	0.02	0.01	0.02	0.16	0.06	0.02	0.22^†^	−0.05	0.12	0.17	0.20
	S.C	0.54^†^	0.48^†^	−0.12	0.04	0.48^†^	0.63^†^		0.82^†^	0.11	0.21^*^	0.07	−0.10	0.21^*^	0.27^*^	0.16	0.10	0.18	0.00	0.31^†^	0.19	0.09
	R.C	0.33^*^	0.21	−0.23	0.19	0.46^†^	0.37^*^	0.82^†^		0.29^*^	0.33^†^	0.06	0.03	0.11	0.17	0.16	0.03	0.21	−0.02	0.33^†^	0.13	0.07
	S.D	0.24	0.18	−0.20	0.10	0.00	−0.01	0.23	0.34		0.52^†^	0.23	0.50^†^	0.03	0.22	−0.08	0.14	0.18	−0.10	0.36^†^	−0.17	0.38^†^
	S.B	0.20	0.23	−0.23	0.12	0.10	0.08	0.29^*^	0.43^†^	0.65^†^		0.29^†^	0.36^†^	0.29^†^	0.26^*^	0.10	0.19	−0.03	0.12	0.77^†^	0.04	0.20
	I.S.B	−0.21	0.23	0.17	0.25	0.00	0.06	0.05	0.18	0.18	0.10		0.05	−0.04	−0.02	0.07	−0.13	−0.05	0.06	0.29^†^	0.05	0.14
	O.S	0.19	0.20	−0.07	0.15	0.05	0.07	0.00	−0.02	0.45^†^	0.34^*^	0.01		−0.04	−0.07	0.06	−0.04	0.15	0.04	0.17	0.10	0.33^†^
Parent-report	F.S	0.47^†^	0.35^*^	−0.22	0.13	0.24	0.15	0.24	0.28	0.33^*^	0.23	−0.16	−0.06		0.57^†^	0.19^*^	0.20^*^	0.14	0.01	0.26^†^	−0.06	−0.26^†^
	S.A	0.27	0.46^†^	−0.07	0.17	0.20	0.25	0.25	0.18	0.49^†^	0.30^*^	0.11	0.14	0.65^†^		0.08	0.21^*^	0.33^†^	0.07	0.18	−0.05	−0.05
	F.O	−0.17	−0.06	0.18	0.23	0.05	0.08	0.07	0.14	0.00	0.08	0.05	0.24	0.11	0.10		0.16	0.19^*^	0.18^*^	0.09	0.03	−0.02
	A.B	−0.12	0.03	0.19	0.26	−0.26	−0.20	−0.20	−0.04	0.14	0.03	−0.01	0.00	0.44	0.25	0.21		0.09	0.09	0.14	−0.34^†^	−0.11
	B.I	0.17	0.25	−0.12	0.36^†^	0.33^†^	0.18	0.17	0.33^*^	0.04	0.13	−0.04	0.18	0.37^†^	0.32^†^	0.40^†^	0.11		0.10	0.13	−0.06	0.05
	P.K	0.20	−0.04	−0.02	0.17	0.05	−0.14	0.26	0.36^*^	0.54^†^	0.30^†^	−0.01	0.18	0.33^†^	0.18	0.26^*^	0.62^†^	0.14		0.05	0.09	0.03
	S.B	0.21	0.14	−0.26	−0.04	0.10	0.10	0.31^*^	0.40^*^	0.44^†^	0.73^†^	0.04	0.14	0.23	0.29^*^	0.13	0.16	0.27^*^	0.39^†^		0.06	0.20^*^
	I.S.B	0.10	−0.16	0.05	−0.01	−0.10	−0.06	0.08	0.01	−0.05	−0.04	−0.04	−0.10	−0.13	−0.10	−0.08	−0.53^†^	0.03	−0.44^†^	0.01		0.30^†^
	O.S	0.14	0.07	−0.14	0.00	−0.07	−0.03	0.13	0.01	0.27	0.29^*^	−0.05	0.47^†^	−0.04	0.10	0.24^*^	0.15	0.14	0.39^†^	0.40^†^	−0.06	

Below the diagonal are correlations in Autism Spectrum Disorder group; above the diagonal are correlations in the Typically Developing group

*FS* friendship skills; *SA* social acceptance; *RS* romantic skills; *PO* personal openness; *FO* family openness; *AB* adequate boundaries; *BI* body image; *SE* self-esteem; *SC* perceived social competence; *RC* romantic confidence; *SD* amount of sexual desire; *PK* psychosexual knowledge; *SB* amount of Sexual behavior; *ISB* amount inappropriate sexualized behavior; *OS* online sexual activity

* = <0.05

^†^ = <0.01

### ASD versus TD: Group Comparisons

#### Psychosexual Socialization

Table 7 shows the results regarding the domain psychosexual socialization. Significant differences, based on both self-report and parent-report, between adolescents with ASD and TD adolescents are found on three of the scales (i.e. ‘Friendship skills’, ‘Social acceptance by peers’ and ‘Adequately dealing with boundaries’). Adolescents with ASD have more problems with peers than the TD adolescents. The other scales (i.e. ‘Romantic skills, ‘Family openness about intimacy’ and ‘Personal openness about intimacy’) showed no significant group differences.

**Table 7:** Results of the domain psychosexual socialization measured with TTI

	Parent-report							Self-report						
	*ASD*		*TD*					*ASD*		*TD*				
	*N*_total_	*M (SD)*	*N*_total_	*M (SD)*	*F*	η_p_^2^	*p*	*N*_total_	*M (SD)*	*N*_total_	*M (SD)*	*F*	η_p_^2^	*p*
Friendship skills scale	76	0.96 (0.58)	128	1.71 (0.33)	112.59	0.36	< 0.001*	56	1.29 (0.53)	88	1.68 (0.29)	26.70	0.16	< 0.001*
Social acceptance by peers scale	76	0.74 (0.61)	128	1.61 (0.47)	120.70	0.38	< 0.001*	55	1.19 (0.48)	91	1.58 (0.36)	35.48	0.20	< 0.001*
Perceived romantic competence scale		n.a		n.a	n.a	n.a	n.a	46	0.88 (0.65)	81	0.78 (0.61)	0.58	0.01	0.45
Personal openness about intimacy scale		n.a		n.a	n.a	n.a	n.a	55	0.44 (0.38)	89	0.61 (0.48)	1.79	0.01	0.18
Family openness about intimacy scale	78	1.20 (0.47)	128	1.14 (0.43)	1.43	0.01	0.23	n.a		n.a		n.a	n.a	n.a
Adequately dealing with boundaries scale	71	1.27 (0.51)	125	1.91 (0.18)	152.67	0.44	< 0.001*	n.a		n.a		n.a	n.a	n.a

*TTI* Teen Transition Inventory; *ASD* autism spectrum disorders; *TD* typically developing; η_p_^2^—partial eta squared, *CI* confidence interval; *n.a* not applicable, not a scale in that version of the TTI, thus no analyses. Each model is adjusted for the effect of gender

*Significant after Bonferroni correction

#### Psychosexual Selfhood

Due to the large number of separate items in the psychosexual selfhood domain (*n* = 7 in the parent-report and *n* = 21 in the self-report), only those items which significantly differed between the two groups are reported in Table 8. Significant differences between the adolescents with ASD and their TD peers were found with regard to the scales ‘Sexual knowledge’, ‘Self-esteem’ and ‘Perceived social competence’ (the latter both on the self-report and parent-report). Only parents reported a significant difference on the scale ‘Body image’. No significant differences were found with regard to ‘Romantic confidence’, ‘Social desires’, ‘Romantic desires’ and ‘Sexual preference’ (i.e. hetero- or homosexual love interests).

**Table 8:** Results of the domain Psychosexual Selfhood measured with TTI

	Parent-report								Self-report								
	ASD		TD						ASD		TD						
Scales	N_total_	M (SD)	N_total_	M (SD)		F	η_p_^2^	p	N_total_	M (SD)	N_total_		M (SD)	F		η_p_^2^	p
Body image scale	76	1.06 (0.49)	129	1.40 (0.42)		32.60	0.14	< 0.001*	56	1.39 (0.33)	90		1.43 (0.38)	2.38		0.02	0.13
Perceived social competence scale		n.a		n.a		n.a	n.a	n.a	55	1.24 (0.36)	91		1.56 (0.32)	31.98		0.18	< 0.001*
Amount of sexual desires scale		n.a		n.a		n.a	n.a	n.a	43	0.87 (0.60)	63		0.83 (0.59)	0.70		0.01	0.41
Self-esteem scale		n.a		n.a		n.a	n.a	n.a	55	1.36 (0.29)	90		1.51 (0.26)	19.41		0.12	< 0.001*
Psychosexual knowledge scale	78	1.79 (0.47)	128	2.04 (0.21)		24.95	0.11	< 0.001*	n.a		n.a		n.a	n.a		n.a	

	ASD		TD						ASD		TD						
Separate items	*N*_total_	%	*N*_total_	%	*OR*	95% CI		p	*N*_total_	%	*N*_total_	%			*OR*	95% CI	p
My child feels uncomfortable around a group of teenagers	76	72.4	128	34.4	7.37	[3.58–15.20]		< 0.001*	n.a		n.a				n.a	n.a	n.a
My child feels confident when hanging out with peers	75	69.3	127	93.7	0.14	[.05-0.37]		< 0.001*	n.a		n.a				n.a	n.a	n.a

* TTI* Teen Transition Inventory; *ASD* autism spectrum disorders; *TD* typically developing, η_p_^2^—partial eta squared, *CI* confidence interval, *n.a*. not applicable, not a scale/item in that version of the TTI, thus no analyses. Each model is adjusted for the effect of gender

*Significant after Bonferroni correction

#### Sexual/intimate Behavior

The results regarding Sexual/intimate behavior are shown in Table 9. Again, only items which significantly differed between the ASD group and the TD group were reported in Table 9. When considering self-report, no significant differences were reported; indicating that according to self-report adolescents with ASD functioned similarly in the domain of Sexual/intimate behavior as their TD peers. However, significant differences between the adolescents with ASD and their TD peers were found when considering the parent-reported scales ‘Amount of sexual/intimate behavior’ and ‘Amount of inappropriate sexualized behavior’. These results indicated less experience with typical sexual/intimate behaviors (e.g. French-kissing), and more inappropriate sexualized behaviors in the adolescents with ASD. In addition, parents reported significant differences in some types of sexual/ intimate behaviors, particularly related to allowing and seeking physical contact with family-members or well-known acquaintances, and taking initiative to seek physical contact with less known acquaintances/strangers. No significant differences were found with regard to age of onset for sexual/intimate behaviors (self-report only), online sexual activity, a variety of types of sexual/intimate behavior (see Table 2 for examples) and the length of the current relationship or age of the current partner (both parent and self-report) .

**Table 9:** Results of the domain Sexual/intimate behavior measured with TTI

	Parent-report								*Self-report*							
	*ASD*		*TD*						*ASD*		*TD*					
Scales	*N*_total_	*M (SD)*	*N*_total_	*M (SD)*		*F*	η_p_^2^	*p*	*N*_total_	*M (SD)*	*N*_total_	*M (SD)*	*F*	η_p_^2^		*p*
Amount of sexual/intimate behaviour scale^a^	74	0.35 (0.32)	122	0.53 (0.37)		10.78	0.05	0.0012*	50	0.37 (0.33)	88	0.53 (0.37)	4.86	0.04		0.03
Amount of inappropriate sexualized behaviour scale^a^	78	0.23 (0.23)	128	0.05 (0.11)		49.28	0.20	< 0.001*	55	0.04 (0.13)	91	0.04 (0.12)	0.03	0.00		0.85
Online sexual activity scale^a^	72	0.34 (0.36)	123	0.22 (0.28)		1.19	0.01	0.28	57	0.15 (0.19)	91	0.09 (0.15)	1.43	0.01		0.24

Separate items	ASD		TD						ASD		TD					
	*N*_total_	%	*N*_total_	%	*OR*		95% CI	*p*	*N*_total_	%	*N*_total_	%	*OR*	95% CI	*p*	
Difficulty being touched by family	77	75.3	129	32.6	6.32		[3.17–12.61]	<0.001*	56	35.7	91	13.2	3.97	[1.58–9.97]	0.003	
Child takes initiative to touch family members	78	65.4	127	93.7	0.10		[.04–0.27]	<0.001*	n.a		n.a		n.a	n.a	n.a	
Child takes initiative to touch less known acquaintances/strangers	78	30.8	127	58.3	0.34		[.18–0.64]	<0.001*	n.a		n.a		n.a	n.a	n.a	

Each row is a separate analysis. *TTI* Teen Transition Inventory; *ASD* autism spectrum disorders; *TD* typically developing, η_p_^2^—partial eta squared, *CI* confidence interval, *n.a*. not applicable; not a scale/item in that version of the TTI, thus no analyses. Each model is adjusted for the effect of gender

*Significant after Bonferroni correction

In our main analyses only gender qualified to be taken into account as a covariate. Although none of the other covariates merited to be taken into account in our analyses, for good measure we additionally ran all the analyses in all three domains including all covariates (i.e. gender, age, IQ and Tanner stages). These analyses yielded very similar results.

## Discussion

Research on psychosexual functioning in individuals with ASD is steadily growing, uncovering more and more information. However, much research has been done with only one informant, not covering all domains of psychosexual functioning or without including a TD control group. Therefore, the current paper aimed to describe the development of comprehensive measure of psychosexual functioning, the TTI, and the initial testing of the TTI. The measure was developed for the purpose of this study, i.e. to compare psychosexual functioning of adolescents with ASD to TD adolescents. Subsequently, we tested the internal consistency of the scales on the TTI and tested whether the TTI distinguished between adolescents with ASD and TD adolescents.

### Measurement Development

The TTI covers all three domains of psychosexual functioning (i.e. psychosexual socialization, psychosexual selfhood, and sexual/intimate behavior) and uses multiple informants. By creating a parent-report and self-report version of the TTI, we were able to get both the perspective of the caregiver as well as the adolescent, which, to our knowledge, was not yet combined in previous studies, highlighting the significance of the current report. During the development of the TTI we took into account results from previous research and attempted to combine constructs into a questionnaire covering all 3 domains of psychosexual functioning, while also making the questionnaire suitable for multiple informants. Of the 9 parent-report and 12 self-report psychosexual scales of the TTI, that were theoretically constructed, most (89% of the parent-report and 83% of the self-report) showed moderate to good internal consistency (Kline 1999; Ponterotto and Ruckdeschel 2007). Two scales (i.e. ‘Amount of inappropriate sexualized behavior’ and ‘Personal Openness about intimacy’) had low internal consistency. The scale ‘Amount of inappropriate sexualized behavior’ measures behavior which is fairly uncommon and thus has a low variance. This may have led to a low internal consistency, which is in line with previous research (e.g. Ginevra et al. 2015; Stokes and Kaur 2005). The other scale (‘Personal openness about intimacy’) measures 1 concept, but the questions cover different aspects of that concept (i.e. feeling comfortable in discussion versus initiating discussion versus discussion in different social groups), which could have led to a lower internal consistency. Correlations showed that there are significant correlations between the several domains of psychosexual functioning, underlining the interrelatedness of its aspects. Often when discussing sexuality, most attention is devoted to the domain of sexual/intimate behavior. However, based on the correlation analyses and previous studies, the domains of psychosexual socialization and psychosexual selfhood are important elements of psychosexual functioning, probably forming the basis on which partnered sexualized/intimate behaviors can be built. The significant correlations between informants imply that at least to some extent informants agree, although no result yielded a perfect correlation. Further research on informant agreement is warranted.

### ASD versus TD

The final aim of this study was to explore differences in psychosexual functioning (i.e. psychosexual socialization, psychosexual selfhood, and sexual/intimate behavior) between adolescents with ASD and TD adolescents. We found significant differences in all domains of psychosexual functioning (i.e. psychosexual socialization, psychosexual selfhood and sexual/intimate behavior) between adolescents with ASD and TD adolescents. Our results showed that in the domain of psychosexual socialization and psychosexual selfhood differences were found both on self-report and parent-report, however in the domain of sexual/intimate behavior, differences were only found when using parent-report. This suggests that, particularly in the domain of sexual/intimate behavior, the results of studies regarding psychosexual functioning may depend on which informant is chosen. Below we discuss our findings per domain of psychosexual functioning in more detail in light of the existing literature.

#### Psychosexual Socialization

According to both parent- and self-report, adolescents with ASD had significantly less friendship skills, and were significantly less accepted by their peers than TD adolescents. The reduced friendship skills and peer relations are in line with previous research regarding social skills (e.g. Mack et al. 2010), which indicated that having ASD is a unique contributing factor to peer relationship problems. Difficulties in this area may become increasingly problematic when reaching adolescence (Murphy and Young 2005), as peer relationships become increasingly important (La Greca and Harrison 2005) and complex (Laugeson et al. 2012). The difficulties with intimacy as well as rejection by peers which adolescents with ASD reported may lead to loneliness, social anxiety and depression (Eussen 2015; La Greca and Harrison 2005) as well as frustration (Hellemans et al. 2010) which have been related to problems such as anxiety and sexual delinquency (Marshall 2010). In addition, due to the problems with peers that the adolescents with ASD and their parents reported, adolescents with ASD may be less able, or less frequently in the position to learn from their peers or other informal social sources (Brown-Lavoie et al. 2014; Stokes et al. 2007; Sullivan and Caterino 2008). This in turn may lead to other problems in psychosexual functioning, which is supported by the correlations we found between the three domains of psychosexual functioning. For example more peer problems is directly correlated with more problems in social competence and romantic confidence, which in turn are directly related to sexual/intimate behavior. These results suggest that targeting more general social skills could be a prerequisite for optimal psychosexual functioning, and thus an improtant target when providing psychosexual guidance or training.

According to parents, adolescents with ASD were also poorer in adequately dealing with boundaries, of both others and oneself. In TD adolescents, experimenting with boundaries is a risk factor to become either the victim or a perpetrator of inappropriate and unwanted sexual/intimate behavior (de Bruijn et al. 2006). Although several studies have looked into inappropriate behaviors or victimization of individuals with ASD, few have investigated the link of being able to deal with boundaries to such behaviors and experiences. In individuals with ASD, the problems with boundaries may be less related to ‘typical’ experimentation but rather a problem to recognize and respect boundaries.

The parents of adolescents with ASD reported similar family openness about psychosexual topics compared to the parents of TD adolescents. Similar to our results, Stokes et al. (2007) found that in cognitively able individuals with ASD (aged 13 to 36 years old) do not significantly differ in learning about social or romantic relationships from their parents in comparison to TD adolescents. Conversely, other research found that parents of children with ASD reported to not find the discussion of sexual or intimate topics relevant, or to be apprehensive to communicate about sexuality topics due to worries, e.g. fear that the child might develop fixation on the topic (Ballan 2012). However, the study of Ballan (2012) was in a group of children with ASD, aged between 6 and 13 years old, whereas our study involved adolescents between the ages of 13 and 21. Potentially when the children with ASD age into adolescence, the parents become more comfortable discussing the topic of sexuality and find it more appropriate. Also, our study took place in a different country with probably different cultural values regarding sexuality. The effect of parent–child communication on risk behaviors in TD samples is mixed (e.g. Clark and Shields 1997; Somers and Paulson 2000), indicating no relation with risk behaviors, and other research indicating reduced or delayed sexual behavior, including sexual risk behaviors (Somers and Paulson 2000) and less delinquency (Clark and Shields 1997). These differences in findings of the effect of communication regarding psychosexual topics may be related to the content discussed by the various sources for example the expectations of the parents (Holmes et al. 2015) and/or the other learning opportunities individuals have.

In our study, adolescents with ASD reported similar personal openness about psychosexual topics as their TD peers. However, in the previously described study of Stokes et al. (2007) it was found that individuals with ASD make significantly fewer use of their peers and friends for information than their TD peers. Possibly due to problems with peers, as found in several studies including our own, adolescents with ASD may experience less opportunity to learn from friends and peers (Brown-Lavoie et al. 2014; Stokes et al. 2007; Sullivan and Caterino 2008).

As parent and peer communication regarding sexuality differs in terms of content and message (Epstein and Ward 2008), different sources may also lead to different results with regard to psychosexual functioning. As our results show similar personal and family openness, but at the same time also difficulties in the various domains of psychosexual functioning, this may mean there is another explanation. Possibly it is not the *quantity* but rather the *content* of the communication which determines whether or not difficulties in psychosexual functioning arise.

#### Psychosexual Selfhood

No significant differences were found with regard to sexual preference. There is both research which supports this (Kellaher 2015) as well as research which found significant differences in sexual preference (Byers et al. 2013). In the study of Byers et al. (2013), adults were used as opposed to adolescents, and participants were directly asked if they identified with a specific sexual orientation. One potential reason for the contradicting finding may be that we did not directly ask the adolescents what their sexual preference was, but rather asked if they had ever been in love with a boy, and if they had ever been in love with a girl, and afterwards based on the adolescents gender recoded this to reflect hetero or homosexual love interests. Our method of assessment of sexual preference reduces the difficulties that individuals may have with the associations of the predefined labels of sexual preference. Yet, we should also note that differences in gender distributions across studies may also explain differences in findings. In the current study, only 14% of the ASD sample was female. Thus, more research is needed to elucidate gender-specific differences in psychosexual functioning in the context of ASD.

In addition, no significant differences were found with regard to social, romantic and amount of sexual desires. This is also in line with previous research (Dewinter et al. 2014; Gilmour et al. 2012; Hénault 2006).

Parents reported a significant differences with regard to the body image of their child, although the adolescents themselves did not report this. Interestingly, little research into body image in individuals with ASD has been conducted. Often it is thought that individuals with ASD may be less influenced by social conventions, as they may be less sensitive to social disapproval (e.g. Ray et al. 2004), which would imply a body image less influenced by social conventions. Potentially, the discrepancy in our study between parent-report and self-report can be attributed to this phenomena; the adolescents themselves are less bothered by social conventions and thus report similar satisfaction with their physical appearance as TD adolescents, whereas parents may be more aware of the discrepancy of the physical appearance of their child with the social conventions, thus reporting a poorer body image. Contrary to our study, Hénault (2006) found that individuals with Asperger syndrome reported a poor body image compared to the general population, implying they are aware of their looks and care about how they look. More research is needed to clarify the body image of individuals with ASD.

Adolescents with ASD and their parents reported lower perceived social competence. This is in line with previous research of Williamson, Craig, and Slinger (2008) as well as the work of Stokes et al. (2007) who found low social competence in adolescents with ASD. Higher perceived social competence is in TD individuals related to spending more time with someone of the other sex (Zimmer-Gembeck and Gallaty 2006). Possibly the lower social competence in adolescents with ASD may also influence the time spend with peers of the other sex, thus limiting romantic and sexual opportunities as well as learning opportunities. Possibly, overall psychosexual functioning in adolescents with ASD could be improved if support is targeted also at the more general social skills.

Surprisingly, the two groups did not differ in their romantic confidence. Some studies have underlined that greater romantic confidence is expected in those with greater social competence (e.g. Giordano et al. 2006), as they may have more experience and thus more comfort with navigating relationships (e.g. experience with making up with a friend could improve confidence in romantic relationships). More research is needed to investigate romantic confidence in adolescents with ASD compared to TD adolescents.

According to parent-report, adolescents with ASD had significantly less psychosexual knowledge (e.g. regarding reproductive health) than TD adolescents. Lack of psychosexual knowledge has the risk of leading to inappropriate sexualized behaviors (Collins et al. 2004). For example the lack of insight in public or private behaviors (Nichols and Blakeley-Smith 2009) or being unable to identify abusive behavior (Sevlever et al. 2013). Specifically in individuals with ASD the level of actual knowledge was found to be a risk factor for sexual victimization (Brown-Lavoie et al. 2014). Our correlations show a direct significant relationship between psychosexual knowledge inappropriate sexualized behavior and difficulties with adequately dealing with boundaries in the ASD group. Although correlations do not provide causal information, this may imply that the improvement of knowledge could lead to improvements in both dealing with boundaries and less inappropriate behavior. One explanation for the limited psychosexual knowledge in the adolescents with ASD may be their particular learning skills; as implicit learning of social cues is weakened in adolescents with ASD (Hudson et al. 2012). Therefore, adolescents with ASD often need to be explicitly taught about psychosexual topics (Gougeon 2010; Hatton and Tector 2010; Sullivan and Caterino 2008). Furthermore, although currently a gradual shift in emphasis and form of sex education at schools is taking place, often sexual education still mainly focuses on the mechanical parts of sexuality rather than the psychosexual aspects. This is probably because in TD adolescents, much knowledge on psychosexual topics is often learned naturally, without explicit teaching, through peers (Andrew et al. 2003; Brown-Lavoie et al. 2014; Stokes et al. 2007). In addition, informal non-social sources (e.g. media and internet) which individuals with ASD use more often (Brown-Lavoie et al. 2014), may provide an incorrect, overly romantic picture of psychosexual functioning or distorted viewpoint through pornography, which could lead to incorrect or limited actual psychosexual knowledge. Therefore it would be valuable to increase the psychosexual knowledge of individuals with ASD, in an attempt to improve the other elements of psychosexual functioning.

#### Sexual/intimate Behavior

In this domain, parents reported significant differences, while adolescents self-reports did not reveal such group differences with regard to both appropriate sexual behavior and inappropriate sexualized behavior. This finding underlines the usefulness of using multiple informants with regard to psychosexual functioning in individuals with ASD.

Parents of adolescents with ASD reported less sexual behavior of their child than the parents of the TD adolescents. The adolescents themselves did not report a significant difference, although they report a similar trend. Both parents and the adolescents indicate that adolescents with ASD have shown approximately one-third of the sexual behaviors and the TD adolescents about half of the sexual behaviors. The non-significant difference found in self-report is in line with other research using self-report, which has found that sexual behavior does not differ between adolescents with ASD and those without ASD (Dewinter et al. 2014). Research in adults using self-report has found that particularly partnered behavior is less common than solitary sexual behavior (i.e. masturbation) when reporting about the last month (Byers et al. 2013), but did not compare this to TD adults. The fact that the adolescents with ASD report slightly higher score on the scale of sexual behaviors than their parents, may be because most sexual behavior is displayed in private (Dewinter et al. 2014) and thus parents may be less aware. In addition, parents of adolescents with ASD may assume that the behavior is non-existent in line with the idea previously explained that parents of individuals with ASD view their children as a-sexual or the topic of sexuality not relevant (Ballan 2012). At the same time, parents of TD adolescents may assume their child does have the experience based on their age rather than factual knowledge. Therefore particularly when investigating psychosexual behavior it is essential to use multiple informants, as different informants may lead to different results and conclusions.

Parents also reported that adolescents with ASD exhibited significantly more inappropriate sexualized behaviors than their TD peers. However it must be noted that the scale of ‘Inappropriate sexualized behavior’ had low internal consistency, most likely due to the low rates of occurrence of the inappropriate behaviors in our sample. Therefore, the results should be interpreted in this light, showing that many of the adolescents with ASD in our sample showed very little to no inappropriate behavior, albeit significantly more than TD adolescents. As has been underlined by previous research, for example that of Demb and Pincus (1993), Ginevra et al. (2015), Hellemans et al. (2007), Kellaher (2015) and Stokes et al. (2007), a diverse range of inappropriate behaviors (e.g. sexually provocative talk, openly discussing sexuality, as well as public masturbation) is reported in adolescents with ASD, regardless of cognitive capacities. Many of these studies however reflect only case-studies or small samples (for a discussion see Kellaher 2015). Our study using a much larger sample of cognitively able adolescents with ASD thus increases our understanding that in fact inappropriate behavior is significantly more likely to occur in individuals with ASD. Even if the behaviors do not escalate to sexual offending, adolescents with ASD who display inappropriate behaviors such as stalking, are at risk for negative outcomes such as criminalization of their behaviors (Stokes et al. 2007) regardless of the intent of their behavior. The findings regarding inappropriate behavior also underlines the importance of using multiple informants.

When looking at stand-alone items, only parents reported difficulties with intimacy with family members and well-known acquaintances both in receiving and initiating it. Such difficulties may complicate the development of intimate and romantic relationships (Tarnai and Wolfe 2008). Although the adolescents themselves do not report these problems, intimacy problems with family may be related to later intimacy problems. Again using multiple informants is important to uncover a better insight in the psychosexual functioning of adolescents with ASD.

### Limitations and Methodological Considerations

Some caution is required when interpreting our results. As the TTI is newly developed, more research should be carried out to investigate the reliability and validity of the inventory. Due to our sample size, we could not perform (confirmatory) factor analyses for the scales as we were underpowered (due to the ratio of items versus participants). This means that the scales were developed based on the literature and expert opinion, and we used statistical analyses to check the Cronbach’s alpha of the scales, which showed that most scales met the criteria for internal consistency. However, some items did not correlate in both samples above the 0.3 limit with the rest of the items on the particular scale (Field 2013), suggesting the scales may be better if these items were removed. Studies with larger samples are on their way to further investigate which items should be removed from the scales. As the TTI is a fairly long measure, it could benefit from item reduction to improve its usability. One way to do this would be to remove the opening questions on general puberty issues and focus it to a psychosexual functioning scale. Also, validation of the TTI against other domain-specific measures is needed. A reliable and validated inventory of psychosexual functioning may improve the comparability and replicability of data and the research performed into this topic. In addition, the results represent a mostly cognitively able group of adolescents with ASD, as the majority of our ASD sample had an IQ within the normal range (scores between 85 and 115). Potentially these cognitively able adolescents may be more aware of their possible difficulties in psychosexual functioning (e.g. social interaction), as particularly cognitively able adolescents with ASD may be aware of their shortcomings in comparison less cognitively able adolescents with ASD (e.g. Locke et al. 2010). Therefore our results may be less applicable to adolescents whom are less cognitively able. Although our pilot indicated that all adolescents were able to read and answer the questionnaire on their own, the reading level of the TTI should be further investigated, especially, when considering using it with less cognitively able participants. Moreover, in the ASD sample, 14% (n = 11) was female, while in the TD sample, this was 54% (n = 71). Although gender was taken into consideration as a covariate in all analyses, clearly, more research is needed to disentangle important gender differences in psychosexual functioning in the context of ASD. Lastly, besides the differences in psychosexual functioning that we found between adolescents with ASD and their TD peers, there were also several 0-findings (for example in the domain sexual/intimate behavior: e.g. age of onset). However, as we used the Bonferroni correction for multiple testing, which is rather conservative and causes some loss of power (increasing chances of Type II error) (Narum 2006), this may mean that in fact some 0-findings are not 0-findings. Therefore, we encourage future research to investigate psychosexual functioning in larger samples to increase power to examine the factor structure of the TTI and replicate our positive as well as 0 findings.

### Strengths

Our study also has three notable strengths. First, we attempted to develop a comprehensive measure including all three domains of psychosexual functioning (i.e. psychosexual socialization, psychosexual selfhood and sexual/intimate behavior). By including all three domains in our inventory we were able to get a more well-rounded view of the level of psychosexual functioning of adolescents with ASD, going beyond only information on a behavioral level. This is particularly important as the interpersonal and intrapersonal domains may form the basis of the behavioral domain. Second, by including a TD comparison group and their parents we were able to directly compare the groups in their psychosexual functioning. Third, as we used both parent-report and self-report we were able to get both perspectives on several aspects of psychosexual functioning of the adolescents. Our results showed that parents and the adolescents themselves did not perceive their psychosexual functioning completely the same, particularly in the domain of sexual behavior (e.g. inappropriate sexualized behavior) and in the domain psychosexual selfhood (i.e. body image). Parents of adolescents with ASD reported significantly more inappropriate sexualized behaviors and a more negative body image than the parents of the TD adolescents, while self-reports did not show significant differences. There are several possible explanations for this difference in parent and self-report: Possibly, parents pathologize the behaviors of their children more, or the child has less self-reflection decreasing the reliability of their self-report. Although parent and self-report showed significant correlations, more research is needed on informant-agreement. Previous research should be considered in the light of which informant was used, as results may differ depending on the informant.

## Overall Conclusions and Implications

Our findings reflect less favorable psychosexual functioning in adolescents with ASD, although the TTI clearly does require more rigorous investigation to both assess its quality and in order to revise the instrument. As could be expected, as social impairments are a hallmark of ASD, adolescents with ASD showed less psychosexual socialization (e.g. on social acceptance, friendship skills and adequately dealing with boundaries) and poorer psychosexual selfhood than their TD peers (i.e. less self-esteem, less social competence and less psychosexual knowledge), both on self-report and parent-report. In the domain of sexual/intimate behavior only significant differences were found on the parent-report, implying less typical and more inappropriate sexualized behaviors as reported by the parents. The interpersonal (socialization) and intrapersonal (selfhood) domains, which significantly correlate with one another, may form a basis for healthy overall psychosexual functioning. Thus difficulties in these domains may underlie difficulties in the behavioral domain. This could explain why earlier studies focused primarily on problematic or inappropriate behaviors, as these were most likely the most pressing and problematic for the environment (e.g. Dewinter et al. 2013; Hellemans et al. 2007; Sevlever et al. 2013; Stokes and Kaur 2005; t Hart-Kerkhoffs et al. 2009). In addition, informants differ in their report of psychosexual functioning, implying the value of using multiple informants. Future research into psychosexual functioning should include multiple informants to aim to get the full picture as well as investigate the factors which may be related to discrepancies between informants.

Regardless of the exact interplay of the elements of psychosexual functioning, the poorer psychosexual functioning of adolescents with ASD may make them more vulnerable also other domains. For example, the poorer psychosexual socialization (e.g. the difficulties with peer relationships) and psychosexual selfhood (e.g. limited self-esteem and limited knowledge) may lead to frustration and loneliness; possibly making adolescents with ASD more vulnerable than their peers (e.g. Brown-Lavoie et al. 2014; de Bruijn et al. 2006; Vickerstaff et al. 2007). Psychosexual training programs may decrease this vulnerability (Kirby et al. 2007) and improve psychosexual functioning, for example by increasing psychosexual knowledge (Dekker et al. 2014). Future research may provide more insight by investigating the interplay between the different domains of psychosexual functioning. This could perhaps shed more light on the differences in psychosexual functioning between individuals with ASD and TD individuals, for example the sources and content of sexual education and their effect on behavior or the effect of self-esteem on social relationships, which potentially could lead to appropriate support in the relevant areas. Also future research could look at how problems in psychosexual functioning in individuals with ASD potentially relates to other domains of their lives. If proper support is provided to improve psychosexual functioning hopefully this could lead to improvements in all aspects of psychosexual functioning and potentially other domains to which it is related.

Many of the psychosexual difficulties that individuals with ASD show may also lead to more extreme difficulties throughout their lives (i.e. sexual victimization or delinquency) (Dennison and Leclerc 2011; Geluk et al. 2012; Markham et al. 2010; Sevlever et al. 2013; Steiger et al. 2014; Trzesniewski et al. 2006). However as the concept is so interrelated investigating only one element may be understating the intricate relations which all contribute to healthy psychosexual functioning (Epstein and Ward 2008). Interventions aimed to improve psychosexual functioning in adolescents with ASD may therefore not only be important to improve psychosexual functioning, but also to decrease the risk of the potential victimization and delinquency.

Although more research is required to determine the quality of the TTI, the differences we found between adolescents with ASD and TD adolescents may support the validity of the TTI as a tool when assessing psychosexual functioning and identifying potential difficulties or problem areas which may need more counseling or help when working with adolescents with ASD. As the literature on psychosexual functioning in adolescents with ASD is still developing and steadily growing, we hope the present study contributes to the understanding of psychosexual functioning in adolescents with ASD and potential difficulties they face. In turn this may contribute to research regarding how to improve psychosexual functioning in adolescents with ASD. Our results indicate that adolescents with ASD have a less favorable psychosexual functioning than TD adolescents in all domains (i.e. psychosexual socialization, psychosexual selfhood and sexual/intimate behavior). Future research could elaborate on our findings to investigate which differences are most prudent to eliminate or minimize and which depend mostly on the informant; to decrease the risks adolescents with ASD have in psychosexual functioning, both with regard to victimization as well as sexual delinquency. Such insights may guide the creation and adaptation of psychosexual training programs aimed to improve psychosexual functioning.

## Electronic supplementary material

Below is the link to the electronic supplementary material.

Supplementary material 1 (DOCX 38 KB)

Supplementary material 2 (DOCX 31 KB)

Supplementary material 3 (DOCX 15 KB)

## References

[CR1] Andrew G, Patel V, Ramakrishna J (2003). Sex, studies or strife? What to integrate in adolescent health services. Reproductive Health Matters.

[CR2] Ballan, M. S. (2012). Parental perspectives of communication about sexuality in families of children with autism spectrum disorders. *Journal of Autism and Developmental Disorders, 42*(5), 676–684. doi:10.1007/s10803-011-1293-y. Retrieved from http://www.embase.com/search/results?subaction=viewrecord&from=export&id=L51482881. 10.1007/s10803-011-1293-y 21681591

[CR3] Baron-Cohen S, Wheelwright S (2003). The Friendship Questionnaire: An investigation of adults with Asperger syndrome or high-functioning autism, and normal sex differences. Journal of Autism and Developmental Disorders.

[CR4] Baron-Cohen S, Wheelwright S, Skinner R, Martin J, Clubley E (2001). The autism-spectrum quotient (AQ): Evidence from asperger syndrome/high-functioning autism, malesand females, scientists and mathematicians. Journal of Autism and Developmental Disorders.

[CR5] Beier ME, Ackerman PL (2003). Determinants of health knowledge: an investigation of age, gender, abilities, personality, and interests. Journal of personality and social psychology.

[CR6] Brown-Lavoie SM, Viecili MA, Weiss JA (2014). Sexual knowledge and victimization in adults with autism spectrum disorders. Journal of Autism and Developmental Disorders.

[CR7] Byers ES, Nichols S, Voyer SD (2013). Challenging stereotypes: Sexual functioning of single adults with high functioning autism spectrum disorder. Journal of Autism and Developmental Disorders.

[CR8] Cederlund M, Hagberg B, Gillberg C (2010). Asperger syndrome in adolescent and young adult males. Interview, self-and parent assessment of social, emotional, and cognitive problems. Research in Developmental Disabilities.

[CR9] Clark, R. D., & Shields, G. (1997). Family communication and delinquency. *Adolescence, 32*(125), 81–92. Retrieved from http://www.scopus.com/inward/record.url?eid=2-s2.0-0031093379&partnerID=40&md5=d15d77f05151973e30beccd3d6abb32e. 9105493

[CR10] Collins RL, Elliott MN, Berry SH, Kanouse DE, Kunkel D, Hunter SB, Miu A (2004). Watching sex on television predicts adolescent initiation of sexual behavior. Pediatrics.

[CR11] Constantino JN, Gruber CP (2007). Social responsiveness scale (SRS).

[CR12] de Bruijn P, Burrie I, van Wel F (2006). A risky boundary: Unwanted sexual behaviour among youth. Journal of Sexual Aggression.

[CR13] de Bruin E, Ferdinand R, Meester S, de Nijs PA, Verheij F (2007). High rates of psychiatric co-morbidity in PDD-NOS. Journal of Autism and Developmental Disorders.

[CR14] Dekker LP, Hartman CA, van der Vegt EJM, Verhulst FC, van Oort FVA, Greaves-Lord K (2014). The longitudinal relation between childhood autistic traits and psychosexual problems in early adolescence: The Tracking Adolescents’ Individual Lives Survey study. Autism.

[CR15] Dekker LP, van der Vegt EJM, Visser K, Tick N, Boudesteijn F, Verhulst FC, Greaves-Lord K (2014). Improving psychosexual knowledge in adolescents with autism spectrum disorder: Pilot of the Tackling Teenage Training Program. Journal of Autism and Developmental Disorders.

[CR16] Demb HB, Pincus SH (1993). Pervasive developmental disorders: A hidden disability in adolescence. Journal of Adolescent Health.

[CR17] Dennis M, Lazenby A, Lockyer L (2001). Inferential language in high-function children with autism. Journal of Autism and Developmental Disorders.

[CR18] Dennison S, Leclerc B (2011). Developmental factors in adolescent child sexual offenders: A comparison of nonrepeat and repeat sexual offenders. Criminal Justice and Behavior.

[CR19] Dewinter, J., Vermeiren, R., Vanwesenbeeck, I., Lobbestael, J., & Van Nieuwenhuizen, C. (2014). Sexuality in adolescent boys with autism spectrum disorder: Self-reported behaviours and attitudes. *Journal of Autism and Developmental Disorders*, 1–11. 10.1007/s10803-014-2226-3 25212415

[CR20] Dewinter J, Vermeiren R, Vanwesenbeeck I, van Nieuwenhuizen C (2013). Autism and normative sexual development: A narrative review. Journal of Clinical Nursing.

[CR21] Dewinter, J., Vermeiren, R. R. J. M., Vanwesenbeeck, I., & Van Nieuwenhuizen, C. (2016). Parental awareness of sexual experience in adolescent boys with autism spectrum disorder. *Journal of Autism and Developmental Disorders*, *46*(2), 713–719. 10.1007/s10803-015-2622-3 PMC4724358 26481386

[CR22] Dorn LD, Susman EJ, Nottelmann ED, Inoff-Germain G, Chrousos GP (1990). Perceptions of puberty: Adolescent, parent, and health care personnel. Developmental Psychology.

[CR23] Epstein M, Ward LM (2008). “Always Use Protection”: Communication Boys Receive About Sex From Parents, Peers, and the Media. Journal of youth and adolescence.

[CR24] Eussen MLJM (2015). Heterogeneity in autism spectrum disorders: Clarifying core and co-occurring characteristics, correlates and course (Doctoral thesis).

[CR25] Evans BE, Greaves-Lord K, Euser AS, Tulen JHM, Franken IHA, Huizink AC (2012). Alcohol and tobacco use and heart rate reactivity to a psychosocial stressor in an adolescent population. Drug and Alcohol Dependence.

[CR26] Falkmer T, Anderson K, Falkmer M, Horlin C (2013). Diagnostic procedures in autism spectrum disorders: A systematic literature review. European Child & Adolescent Psychiatry.

[CR27] Fenton KA, Johnson AM, McManus S, Erens B (2001). Measuring sexual behaviour: Methodological challenges in survey research. Sexually Transmitted Infections.

[CR28] Field AP (2013). Discovering statistics using IBM SPSS Statistics: and sex and drugs and rock ‘n’ roll.

[CR29] Flannery DJ, Rowe DC, Gulley BL (1993). Impact of pubertal status, timing, and age on adolescent sexual experience and delinquency. Journal of Adolescent Research.

[CR30] Geluk CAML, Jansen L, Vermeiren R, Doreleijers TAH, van Domburgh L, de Bildt A, Hartman CA (2012). Autistic symptoms in childhood arrestees: Longitudinal association with delinquent behavior. Journal of Child Psychology and Psychiatry.

[CR31] Gilmour L, Schalomon PM, Smith V (2012). Sexuality in a community based sample of adults with autism spectrum disorder. Research in Autism Spectrum Disorders.

[CR32] Ginevra MC, Nota L, Stokes MA (2015). The differential effects of Autism and Down’s syndrome on sexual behavior. Autism Research.

[CR33] Giordano PC, Longmore MA, Manning WD (2006). Gender and the meanings of adolescent romantic relationships: A focus on boys. American Sociological Review.

[CR34] Gougeon NA (2010). Sexuality and autism: A critical review of selected literature using a social-relational model of disability. American Journal of Sexuality Education.

[CR35] Halpern CT, Udry JR, Campbell B, Suchindran C (1993). Testosterone and pubertal development as predictors of sexual activity: A panel analysis of adolescent males. Psychosomatic Medicine.

[CR36] Hatton S, Tector A (2010). FOCUS ON PRACTICE: Sexuality and relationship education for young people with autistic spectrum disorder: curriculum change and staff support. British Journal of Special Education.

[CR37] Hearn KD, O’Sullivan LF, Dudley CD (2003). Assessing reliability of early adolescent girls’ reports of romantic and sexual behavior. Archives of Sexual Behavior.

[CR38] Hellemans, H., Colson, K., Verbraeken, C., Vermeiren, R., & Deboutte, D. (2007). Sexual behavior in high-functioning male adolescents and young adults with autism spectrum disorder. *Journal of Autism and Developmental Disorders, 37*(2), 260–269. doi:10.1007/s10803-006-0159-1. Retrieved from http://www.embase.com/search/results?subaction=viewrecord&from=export&id=L46311119. 10.1007/s10803-006-0159-1 16868848

[CR39] Hellemans, H., Roeyers, H., Leplae, W., Dewaele, T., & Deboutte, D. (2010). Sexual behavior in male adolescents and young adults with autism spectrum disorder and borderline/mild mental retardation. *Sexuality and Disability, 28*(2), 93–104. doi:10.1007/s11195-009-9145-9. Retrieved from http://www.embase.com/search/results?subaction=viewrecord&from=export&id=L50759906.

[CR40] Hénault I (2006). Asperger’s syndrome and sexuality - From adolescence through adulthood.

[CR41] Holmes LG, Himle MB, Strassberg DS (2015). Parental romantic expectations and parent–child sexuality communication in autism spectrum disorders. Autism.

[CR42] Hudson M, Nijboer TCW, Jellema T (2012). Implicit social learning in relation to autistic-like traits. Journal of Autism and Developmental Disorders.

[CR43] Kasari C, Locke J, Gulsrud A, Rotheram-Fuller E (2011). Social networks and friendships at school: Comparing children with and without ASD. Journal of Autism and Developmental Disorders.

[CR44] Kellaher DC (2015). Sexual behavior and autism spectrum disorders: An update and discussion. Current Psychiatry Reports.

[CR45] Kirby DB, Laris BA, Rolleri LA (2007). Sex and HIV education programs: their impact on sexual behaviors of young people throughout the world. Journal of Adolescent Health.

[CR46] Kline P (1999). Handbook of Psychological Testing.

[CR47] Koller, R. (2000). Sexuality and adolescents with autism. *Sexuality and Disability, 18*(2), 125–135. doi:10.1023/a:1005567030442. Retrieved from http://www.embase.com/search/results?subaction=viewrecord&from=export&id=L30679653.

[CR48] La Greca AM, Harrison HM (2005). Adolescent peer relations, friendships, and romantic relationships: Do they predict social anxiety and depression?. Journal of Clinical Child and Adolescent Psychology.

[CR49] Laugeson EA, Frankel F, Gantman A, Dillon AR, Mogil C (2012). Evidence-based social skills training for adolescents with autism spectrum disorders: The UCLA PEERS Program. Journal of Autism and Developmental Disorders.

[CR50] Lerner MD, Calhoun CD, Mikami AY, De Los Reyes A (2012). Understanding parent–child social informant discrepancy in youth with high functioning Autism spectrum disorders. Journal of Autism and Developmental Disorders.

[CR51] Locke J, Ishijima EH, Kasari C, London N (2010). Loneliness, friendship quality and the social networks of adolescents with high-functioning autism in an inclusive school setting. Journal of Research in Special Educational Needs.

[CR52] Lord, C., Risi, S., Lambrecht, L., Cook, E. H., Jr., Leventhal, B. L., DiLavore, P. C.,…& Rutter, M. (2000). The autism diagnostic observation schedule-generic: a standard measure of social and communication deficits associated with the spectrum of autism. *Journal of Autism and Developmental Disorders, 30*(3), 205–223. Retrieved from http://www.ncbi.nlm.nih.gov/entrez/query.fcgi?cmd=Retrieve&db=PubMed&dopt=Citation&list_uids=11055457. 11055457

[CR53] Louwerse A, Eussen MLJM, Van der Ende J, de Nijs PFA, Van Gool AR, Dekker LP, Greaves-Lord K (2015). ASD Symptom severity in adolescence of individuals diagnosed with pdd-nos in childhood: Stability and the Relation with psychiatric comorbidity and societal participation. Journal of Autism and Developmental Disorders.

[CR54] Louwerse A, van der Geest JN, Tulen JHM, van der Ende J, Van Gool AR, Verhulst FC, Greaves-Lord K (2013). Effects of eye gaze directions of facial images on looking behaviour and autonomic responses in adolescents with autism spectrum disorders. Research in Autism Spectrum Disorders.

[CR55] Mack, H., Fullana, M. A., Russell, A. J., Mataix-Cols, D., Nakatani, E., & Heyman, I. (2010). Obsessions and compulsions in children with Asperger’s syndrome or high-functioning autism: A case-control study. *Australian and New Zealand Journal of Psychiatry, 44*(12), 1082–1088. doi:10.3109/00048674.2010.515561. Retrieved from http://www.embase.com/search/results?subaction=viewrecord&from=export&id=L359984442. 10.3109/00048674.2010.515561 20973622

[CR56] Mandy W, Chilvers R, Chowdhury U, Salter G, Seigal A, Skuse D (2012). Sex differences in autism spectrum disorder: Evidence from a large sample of children and adolescents. Journal of Autism and Developmental Disorders.

[CR57] Markham CM, Lormand D, Gloppen KM, Peskin MF, Flores B, Low B, House LD (2010). Connectedness as a predictor of sexual and reproductive health outcomes for youth. Journal of Adolescent Health.

[CR58] Marshall WA, Tanner JM (1969). Variations in pattern of pubertal changes in girls. Archives of disease in childhood.

[CR59] Marshall WA, Tanner JM (1970). Variations in the pattern of pubertal changes in boys. Archives of disease in childhood.

[CR60] Marshall WL (2010). The role of attachments, intimacy, and loneliness in the etiology and maintenance of sexual offending. Sexual and Relationship Therapy.

[CR61] Martin I, McDonald S (2004). An exploration of causes of non-literal language problems in individuals with Asperger Syndrome. Journal of Autism and Developmental Disorders.

[CR62] Mehzabin P, Stokes MA (2011). Self-assessed sexuality in young adults with high-functioning autism. Research in Autism Spectrum Disorders.

[CR63] Murphy N, Young PC (2005). Sexuality in children and adolescents with disabilities. Developmental Medicine & Child Neurology.

[CR64] Narum SR (2006). Beyond Bonferroni: Less conservative analyses for conservation genetics. Conservation Genetics.

[CR65] Nichols S, Blakeley-Smith A (2009). “I’m Not Sure We’re Ready for This…”: working with families toward facilitating healthy sexuality for individuals with autism spectrum disorders. Social Work in Mental Health.

[CR66] O’Sullivan LF, Cheng MM, Harris KM, Brooks-Gunn J (2007). I Wanna hold your hand: The progression of social, romantic and sexual events in adolescent relationships. Perspectives on Sexual and Reproductive Health.

[CR67] Ponterotto JG, Ruckdeschel DE (2007). An overview of coefficient alpha and a reliability matrix for estimating adequacy of internal consistency coefficients with psychological research measures. Perceptual and Motor Skills.

[CR68] Ray F, Marks C, Bray-Garretson H (2004). Challenges to treating adolescents with asperger’s syndrome who are sexually abusive. Sexual Addiction & Compulsivity.

[CR69] Realmuto, G. M., & Ruble, L. A. (1999). Sexual behaviors in autism: Problems of definition and management. *Journal of Autism and Developmental Disorders, 29*(2), 121–127. Retrieved from http://www.ncbi.nlm.nih.gov/entrez/query.fcgi?holding=inleurlib_fft&cmd=Retrieve&db=PubMed&dopt=Citation&list_uids=10382132, http://ovidsp.ovid.com/ovidweb.cgi?T=JS&CSC=Y&NEWS=N&PAGE=fulltext&D=med4&AN=10382132. 10.1023/a:1023088526314 10382132

[CR70] Rutter M, Le Couteur A, Lord C (2003). Autism diagnostic interview-revised.

[CR71] Sevlever M, Roth ME, Gillis JM (2013). Sexual abuse and offending in autism spectrum disorders. Sexuality and Disability.

[CR72] Shandra CL, Chowdhury AR (2012). The first sexual experience among adolescent girls with and without disabilities. Journal of Youth and Adolescence.

[CR73] Somers CL, Paulson SE (2000). Students’ perceptions of parent–adolescent closeness and communication about sexuality: relations with sexual knowledge, attitudes, and behaviors. Journal of Adolescence.

[CR74] Steiger AE, Allemand M, Robins RW, Fend HA (2014). Low and decreasing self-esteem during adolescence predict adult depression two decades later. Journal of Personality and Social Psychology.

[CR75] Stokes M, Kaur A (2005). High-functioning autism and sexuality: A parental perspective. Autism.

[CR76] Stokes, M., Newton, N., & Kaur, A. (2007). Stalking, and social and romantic functioning among adolescents and adults with autism spectrum disorder. *Journal of Autism and Developmental Disorders, 37*(10), 1969–1986. doi:10.1007/s10803-006-0344-2. Retrieved from http://www.embase.com/search/results?subaction=viewrecord&from=export&id=L47609354. 10.1007/s10803-006-0344-2 17273936

[CR77] Sullivan A, Caterino LC (2008). Addressing the sexuality and sex education of individuals with autism spectrum disorders. Education and Treatment of Children.

[CR78] t Hart-Kerkhoffs LA, Jansen LM, Doreleijers TA, Vermeiren R, Minderaa RB, Hartman CA (2009). Autism spectrum disorder symptoms in juvenile suspects of sex offenses. The Journal of clinical psychiatry.

[CR79] Tarnai B, Wolfe PS (2008). Social stories for sexuality education for persons with autism/pervasive developmental disorder. Sexuality and Disability.

[CR80] Tick NT, Van der Ende J, Verhulst FC (2008). Ten-year increase in service use in the Dutch population. European Child & Adolescent Psychiatry.

[CR81] Tolman DL, McClelland SI (2011). Normative sexuality development in adolescence: A decade in review, 2000–2009. Journal of Research on Adolescence.

[CR82] Trzesniewski KH, Donnellan MB, Moffitt TE, Robins RW, Poulton R, Caspi A (2006). Low self-esteem during adolescence predicts poor health, criminal behavior, and limited economic prospects during adulthood. Developmental Psychology.

[CR83] Urbano MR, Hartmann K, Deutsch SI, Polychronopoulos GMB, Dorbin V, Fitzgerald M (2013). Relationships, sexuality, and intimacy in autism spectrum disorders. Recent advances in autism spectrum disorders.

[CR84] Vickerstaff S, Heriot S, Wong M, Lopes A, Dossetor D (2007). Intellectual ability, self-perceived social competence, and depressive symptomatology in children with High-functioning autistic spectrum disorders. Journal of Autism and Developmental Disorders.

[CR85] Wechsler D (1999). Wechsler abbreviated scale of intelligence, manual.

[CR86] Wechsler D (2004). The Wechsler intelligence scale for children-fourth edition.

[CR87] Whitehouse, A. J. O., Maybery, M. T., Hickey, M., & Sloboda, D. M. (2011). Brief report: Autistic-like traits in childhood predict later age at menarche in girls. *Journal of Autism and Developmental Disorders, 41*(8), 1125–1130. doi:10.1007/s10803-010-1129-1. Retrieved from http://www.embase.com/search/results?subaction=viewrecord&from=export&id=L51205354. 10.1007/s10803-010-1129-1 21184161

[CR88] Williamson S, Craig J, Slinger R (2008). Exploring the relationship between measures of self-esteem and psychological adjustment among adolescents with Asperger Syndrome. Autism: the international journal of research and practice.

[CR89] World Health Organization. (2006). Defining sexual health. Report of a technical consultation on sexual health 28–31 January 2002, Geneva. Retrieved from Geneva: http://www.who.int/reproductivehealth/publications/sexual_health/defining_sexual_health.pdf.

[CR90] Zimmer-Gembeck MJ, Gallaty KJ (2006). Hanging out or hanging in? Young females’ socioemotional functioning and the changing motives for dating and romance. Advances in Psychology Research.

