# Supplementary material for: Psychosexual Functioning of Cognitively-able Adolescents with Autism Spectrum Disorder Compared to Typically Developing Peers: The Development and Testing of the Teen Transition Inventory- a Self- and Parent Report Questionnaire on Psychosexual Functioning
Source: J Autism Dev Disord. 2017 Mar 16;47(6):1716–38. doi: 10.1007/s10803-017-3071-y (PMC5432605; doi:10.1007/s10803-017-3071-y)
Supplement: Supplementary file 1 — Supplementary material 1 (DOCX 38 KB) [file 10803_2017_3071_MOESM1_ESM.docx]

Appendix 1a: Sample of the TTI-adolescent version

**YOU & LOVE**

Below is a list of items that describe adolescents. All these items concern issues on how you are now or have been in the last 6 months. Please answer *very or often true* if this expression is appropriate to your situation. Please answer *somewhat or sometimes true* if this expression is somewhat or sometimes true to your situation. If the expression is not at all appropriate to your situation, please answer *not at all true*.

|  | Not at all true | Somewhat or Sometimes true | Very or Often true |
| --- | --- | --- | --- |
| 1. **I have been in love with someone. (If you choose *not at all* skip all questions up until question 24 and start with question 25 on page 14)** | ○ | ○ | ○ |
| 1. **I have been in love with a peer.** | ○ | ○ | ○ |
| 1. **I have been in love with someone who I know through the Internet.** | ○ | ○ | ○ |
| 1. **I have been in love with a celebrity (= an existing person).** | ○ | ○ | ○ |
| 1. **I have been in love with a fictional character from TV or a computer game (= a fictional person).** | ○ | ○ | ○ |
| 1. **I have been in love with a teacher or mentor or group-leader.** | ○ | ○ | ○ |
| 1. **I have been in love with someone else, namely: _____________________________ _____________________________** | ○ | ○ | ○ |
| 1. **I have been in love with a boy/man.** | ○ | ○ | ○ |
| 1. **I have been in love with a girl/woman.** | ○ | ○ | ○ |
| 1. **I have been in love with someone a lot younger than I was (5 years or more).** | ○ | ○ | ○ |
| 1. **I have been in love with someone a lot older than I was (5 years or more).** | ○ | ○ | ○ |
| 1. **I have been in love with someone although that person was not in love with me.** | ○ | ○ | ○ |
| 1. **I have been in love with someone and that person was also in love with me.** | ○ | ○ | ○ |

**YOU & LOVE**

|  | Not at all true | Somewhat or Sometimes true | Very or Often true |
| --- | --- | --- | --- |
| 1. When I am in love with someone, I feel confident. | ○ | ○ | ○ |
| 1. When I am in love with someone, I do not know what to do. | ○ | ○ | ○ |
| 1. When I am in love with someone, I do not know what to say. | ○ | ○ | ○ |
| 1. When I am in love with someone, I do not know how to make contact with that person. | ○ | ○ | ○ |
| 1. When I am in love with someone, I contact that person using the internet (MSN/Facebook/email/social networks). | ○ | ○ | ○ |
| 1. When I am in love with someone, I start a conversation with that person. | ○ | ○ | ○ |
| 1. When I am in love with someone, I do something else to come into contact with him or her, namely: ­____________________________ ____________________________ ____________________________ | ○ | ○ | ○ |
| 1. The first time I have contact with the person I am in love with, I ask for contact information (= phone number, email address, MSN, Facebook etc). | ○ | ○ | ○ |
| 1. The first time I have contact with the person I am in love with, I ask if he or she wants to meet up with me. | ○ | ○ | ○ |
| 1. The first time I have contact with the person I am in love with, I tell that person I am in love with him or her. | ○ | ○ | ○ |
| 1. The first time I have contact with the person I am in love with, I ask him or her to be my boyfriend/girlfriend. | ○ | ○ | ○ |

|  |  |  |  |
| --- | --- | --- | --- |
| 1. I keep contacting someone, even though that person has indicated he/she does not want any contact with me. | ○ | ○ | ○ |

**SEXUALITY**

During puberty many teenagers are curious about intimacy. Some teenagers will have their experiences with intimacy.

1. This question consists of 3 subparts; carefully read the instructions below before you answer the question.
2. Of the actions named below, please answer if you have ever done that and if you have done it, how old you were when you first did it.
3. After completing part I, please answer if you have done it in the past 6 months.
4. If you have never done it, we ask if you to please answer if you would want to do it.

|  | **I) Ever**  No Yes Age 1^st^  time: | **II) Past six months**  Never Sometimes Often | **III) Would you want to do it**  Not at A little Very  all much |
| --- | --- | --- | --- |
| Masturbating |   ____ |    |    |
| French kissing |   ____ |    |    |
| Touching and caressing |   ____ |    |    |
| Fingering by or of someone / giving or receiving a hand-job |   ____ |    |    |
| Intercourse (making love, sex) |   ____ |    |    |
| Something else, namely:_________________________ |   ____ |    |    |

|  | Not at all true | Somewhat or Sometimes true | Very or Often true |
| --- | --- | --- | --- |
| 1. When I am physically intimate with another person (for example making out, cuddling or sex) I am good at pointing out what I do and do not find pleasant. | ○ | ○ | ○ |
| 1. I fantasize sometimes about being physically intimate with someone (for example French kissing, cuddling or sex). | ○ | ○ | ○ |
| 1. I have had a very unpleasant intimate experience (for example French kissing, cuddling or sex). | ○ | ○ | ○ |
| 1. I find it pleasant to be physically intimate with another person (for example French kissing, cuddling or sex). | ○ | ○ | ○ |
| 1. I feel confident when I am physically intimate (for example French kissing, cuddling or sex) with someone. | ○ | ○ | ○ |
| 1. I do not know how to masturbate (having sex with yourself, touching your private parts/caressing). | ○ | ○ | ○ |

- 1. Do you have a specific physical limitation or at the moment have any physical problems (for example infections or injuries on the genitals) that make having intimate relations or sexuality more difficult?

○ No

○ Yes, such as: ________________________________

**YOU & THE INTERNET**

Below is a list of items that describe adolescents. All these items concern issues on how you are now or have been in the last 6 months. Please answer *very or often true* if this expression is appropriate to your situation. Please answer *somewhat or sometimes true* if this expression is somewhat or sometimes true to your situation. If the expression is not at all appropriate to your situation, please answer *not at all true*.

|  | Not at all true | Somewhat or Sometimes true | Very or Often true |
| --- | --- | --- | --- |
| 1. I make use of the Internet. | ○ | ○ | ○ |
| 1. I have contact on the Internet with people whom I know from my immediate surroundings (for example, chatting, and social networks). | ○ | ○ | ○ |
| 1. I have contact on the Internet with people who I do NOT know before I first contacted them using the Internet (for example, chatting, and social networks). | ○ | ○ | ○ |
| 1. I have contact with people using a webcam. | ○ | ○ | ○ |
| 1. I visit websites that give information about sex. | ○ | ○ | ○ |
| 1. I visit websites with sexual imagery or movies (=porno). | ○ | ○ | ○ |
| 1. I have set a date with someone I met on the Internet. | ○ | ○ | ○ |
| 1. I have had sex with someone I met on the Internet. | ○ | ○ | ○ |
| 1. I have shown myself naked via the webcam. | ○ | ○ | ○ |
| 1. Another person has shown himself/herself naked via the webcam to me. | ○ | ○ | ○ |
| 1. I have had sex with someone via the Internet (=cybersex). | ○ | ○ | ○ |
