# Supplementary material for: Psychosexual Functioning of Cognitively-able Adolescents with Autism Spectrum Disorder Compared to Typically Developing Peers: The Development and Testing of the Teen Transition Inventory- a Self- and Parent Report Questionnaire on Psychosexual Functioning
Source: J Autism Dev Disord. 2017 Mar 16;47(6):1716–38. doi: 10.1007/s10803-017-3071-y (PMC5432605; doi:10.1007/s10803-017-3071-y)
Supplement: Supplementary file 2 — Supplementary material 2 (DOCX 31 KB) [file 10803_2017_3071_MOESM2_ESM.docx]

**Appendix 1b Sample of the TTI parent version**

**YOUR CHILD & SEXUAL EDUCATION**

1. Has your child already had sexual education or training on the topic of puberty/friendship/intimacy/sexuality?

○ No

○ Yes 🡪 where/from whom did your child receive sexual education? You are allowed to give more than one answer.

○ By his parents/caregivers

○ By his teacher at school

○ By his friends/peers

○ By the media and/or the internet

○ At a special training, namely ______________________________________________ ______________________________________________

○ Other, namely: ______________________________________________ ______________________________________________

**Family**

Every family has their own habits and ways of doing things. In the one family there is more openness about the naked body or discussing sexuality than there is in other families. The following questions centre around the habits of your family.

Below is a list of items. Each item concerns how your child is now or has been in the last 6 months. Please answer Very or often true if this expression is appropriate to your child’s situation. Please answer somewhat or sometimes true if this expression is somewhat or sometimes true to your child’s situation. If the expression is not at all appropriate to your child’s situation, please answer not at all true.

|  | Not at all true | Somewhat or Sometimes true | Very or Often true |
| --- | --- | --- | --- |
| 1. Within our family we discuss sexuality | ○ | ○ | ○ |
| 1. We/I as parent(s) are comfortable talking about sexuality with the child for whom we are filling out the questionnaire. | ○ | ○ | ○ |
| 1. My child takes the initiative to talk about sexuality with me/us. | ○ | ○ | ○ |
| 1. We/I as parent(s) take the initiative to talk about sexuality. | ○ | ○ | ○ |

**Knowledge**

Some teenagers have a limited knowledge about physical development and sexuality, other teenagers know a lot about it. The following questions are focused on the knowledge your child has about these subjects.

|  | Not at all true | Somewhat or Sometimes true | Very or Often true |
| --- | --- | --- | --- |
| 1. My child has knowledge about the physical changes that occur during puberty. | ○ | ○ | ○ |
| 1. My child is able to name his/her body parts. | ○ | ○ | ○ |
| 1. My child knows the correct meaning of the sexual terminology he/she uses. | ○ | ○ | ○ |
| 1. My child has knowledge about the different types of sexual preference that exist (hetero-, homo-, bisexual). | ○ | ○ | ○ |
| 1. My child knows how a woman can become pregnant. | ○ | ○ | ○ |
| 1. My child knows what precautions to take to prevent pregnancy. | ○ | ○ | ○ |
| 1. My child knows how babies are conceived. | ○ | ○ | ○ |
| 1. My child knows people have intercourse and how they do that. | ○ | ○ | ○ |
| 1. My child knows what precautions to take to prevent getting a venereal disease/sexually transmitted disease (STD). | ○ | ○ | ○ |

**YOUR CHILD & INTIMACY**

**Intimacy**

Physical contact is commonly made during friendly contact. Some teenagers have trouble with this, for example with being touched and touching others.

Below is a list of items. Each item concerns how your child is now or has been in the last 6 months. Please answer Very or often true if this expression is appropriate to your child’s situation. Please answer somewhat or sometimes true if this expression is somewhat or sometimes true to your child’s situation. If the expression is not at all appropriate to your child’s situation, please answer not at all true.

|  | Not at all true | Somewhat or Sometimes true | Very or Often true |
| --- | --- | --- | --- |
| 1. My child takes initiative to touch other family members or well-known acquaintances. | ○ | ○ | ○ |
| 1. My child has difficulty with being touched by other family members or well-known acquaintances. | ○ | ○ | ○ |
| 1. My child takes the initiative to touch less known acquaintances/strangers. | ○ | ○ | ○ |
| 1. My child has difficulty with being touched by acquaintances/strangers. | ○ | ○ | ○ |
| 1. My child touches others where they do not like to be touched. | ○ | ○ | ○ |
|  |  |  |  |
| 1. My child realizes it when he/she is touching people inappropriately. | ○ | ○ | ○ |

1. Who does your child touch inappropriately?

○ Family members/acquaintances

○ Friends

○ Strangers

○ Other, such as: ________________________________

|  | Not at all true | Somewhat or Sometimes true | Very or Often true |
| --- | --- | --- | --- |
| 1. My child follows the social rules that are accepted in the different situations that centre around nudity (for example changing with the curtains drawn). | ○ | ○ | ○ |
| 1. My child poses questions or makes remarks about intimate topics that are socially inappropriate (for example asking (too) personal questions or making personal remarks at inappropriate times). | ○ | ○ | ○ |
| 1. My child is able to set his/her boundaries regarding social relationships in general. | ○ | ○ | ○ |
| 1. My child is able to recognize other people’s boundaries regarding social relationships in general. (This means your child is able to recognize another person’s non-verbal-hints) | ○ | ○ | ○ |
| 1. My child responds adequately to other people’s boundaries regarding social relationships in general. (Your child adjusts his/her behavior depending on what the other person indicates) | ○ | ○ | ○ |
| 1. I worry about the defensibility of my child regarding social relationships in general. | ○ | ○ | ○ |
