# Supplementary material for: Psychosexual Functioning of Cognitively-able Adolescents with Autism Spectrum Disorder Compared to Typically Developing Peers: The Development and Testing of the Teen Transition Inventory- a Self- and Parent Report Questionnaire on Psychosexual Functioning
Source: J Autism Dev Disord. 2017 Mar 16;47(6):1716–38. doi: 10.1007/s10803-017-3071-y (PMC5432605; doi:10.1007/s10803-017-3071-y)
Supplement: Supplementary file 3 — Supplementary material 3 (DOCX 15 KB) [file 10803_2017_3071_MOESM3_ESM.docx]

**Appendix 2** ‘item-rest correlations below .3’

Fifteen items (six items on parent-report scales and nine items in the self-report scales) showed an item-rest correlation below .3 in both samples: 1 item on the scale ‘Body image’ (self-report); 1 item on the scale ‘Personal openness about intimacy’ (self-report); 3 items on the scale ‘Self-esteem’(self-report); 1 item on the scale ‘Amount of sexual behavior’ (both parent-report and self-report); several items on the scale ‘Amount of inappropriate sexualized behavior’(parent-report = 4 items and self-report = 3 items); and 1 item on the scale ‘Online sexual activity’ (parent-report) had an item-rest correlation below .3 (detailed information available upon request).

Eight of the 15 items with a low item-rest correlation are part of the two scales which showed low internal consistency (i.e. ´Amount of inappropriate sexualized behavior´ and ‘Personal Openness about intimacy’). With regard to the ´Amount of inappropriate sexualized behavior´ scale, this scale showed low internal consistency on both parent-report and self-report in both groups (ASD and TD), and in the TD self-report even a negative Cronbach’s alpha. Although the low alpha’s of the ‘Amount of inappropriate sexualized behavior’ would generally be worrisome, the behaviors reported on in this scale range from rather moderate to extremely inappropriate behaviors (see Table 2 for example questions) thus it can be expected that the more extreme behaviors are sparsely endorsed by our non-criminal groups. Similar findings with regard to sexual behavior have been found in other studies; i.e. the studies of [Ginevra et al. (2015)](#_ENREF_32) and of [Stokes and Kaur (2005)](#_ENREF_75). Specifically, in the parent-report scale, the item endorsement of the 4 items with low item-rest correlation range from 0% (i.e. inappropriate masturbation) to 8.1% (i.e. inappropriately taking care of personal hygiene surrounding either menstruation or ejaculation) in the TD group. In the ASD group the endorsement was somewhat higher, ranging from 2.1% (i.e. inappropriate masturbation) to 46.9% (i.e. inappropriately taking care of personal hygiene surrounding either menstruation or ejaculation). This explains why the Cronbach’s alpha was also better in the ASD group. The 3 items on the self-report scale of ´Amount of inappropriate sexualized behavior´ were sparsely endorsed in both the TD group (ranges from 2.2% to 6.6%) and the ASD group (ranges from 3.6 to 7.1%). The low internal consistency on the Openness scale may be because the items on this scale measure different aspects of openness (i.e. communication regarding sexuality with different people), potentially thereby decreasing the Cronbach’s alpha.

The remaining 7 items were checked on their content validity and effect on the Cronbach’s alpha. All items suited the scale they are part of based on content, but may not have correlated optimally with the other items in the scale as they measure (slightly) different aspects of the construct measured with the full scale. For example, the item in sexual behavior with an item-rest correlation below 0.3 in both samples asked about masturbation behavior, which is a solo-sexual activity, whilst the other items covering sexual behavior are at least partnered sexual activities.
